# PKC-independent PI3K signalling diminishes PKC inhibitor sensitivity in uveal melanoma

**DOI:** 10.1038/s41389-024-00511-8

**Published:** 2024-02-28

**Authors:** John J. Park, Sabine Abou Hamad, Ashleigh Stewart, Matteo S. Carlino, Su Yin Lim, Helen Rizos

**Affiliations:** 1 Macquarie Medical School, Faculty of Medicine, Human and Health Sciences, Macquarie University, Sydney, NSW Australia; 2 Melanoma Institute Australia, The University of Sydney, Sydney, NSW Australia; 3 Department of Medical Oncology, Westmead and Blacktown Hospitals, Sydney, NSW Australia

**Keywords:** Cancer models, Apoptosis

## Abstract

Protein kinase C (PKC) is activated downstream of gain-of-function *GNAQ* or *GNA11* (*GNAQ/GNA11*) mutations in over 90% of uveal melanoma (UM). Phase I clinical trials of PKC inhibitors have shown modest response rates with no survival benefit in metastatic UM. Although PKC inhibitors actively suppress mitogen-activated protein kinase (MAPK) signalling in UM, the effect on other UM signalling cascades is not well understood. We examined the transcriptome of UM biopsies collected pre- and post-PKC inhibitor therapy and confirmed that MAPK, but not PI3K/AKT signalling, was inhibited early during treatment with the second-generation PKC inhibitor IDE196. Similarly, in GNAQ/GNA11-mutant UM cell models, PKC inhibitor monotherapy effectively suppressed MAPK activity, but PI3K/AKT signalling remained active, and thus, concurrent inhibition of PKC and PI3K/AKT signalling was required to synergistically induce cell death in a panel of GNAQ/GNA11-mutant UM cell lines. We also show that re-activation of MAPK signalling has a dominant role in regulating PKC inhibitor responses in UM and that PI3K/AKT signalling diminishes UM cell sensitivity to PKC inhibitor monotherapy. Thus, combination therapies targeting PKC and PKC-independent signalling nodes, including PI3K/AKT activity, are required to improve responses in patients with metastatic UM.

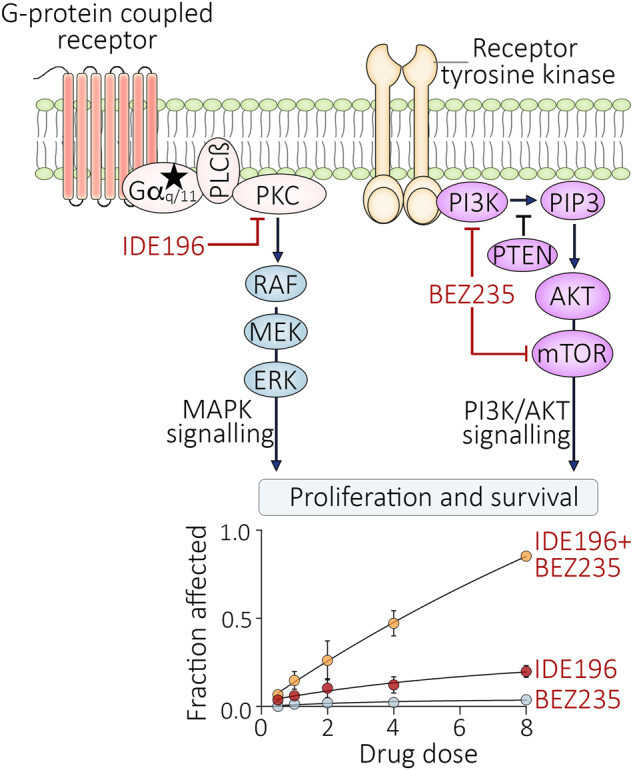

## Introduction

Uveal melanoma (UM) is the most common primary ocular malignancy in adults, with an incidence of 5–7 individuals per million per year. Despite favourable local outcomes after primary UM treatment, the long-term prognosis of UM remains poor, with up to 50% of patients developing metastatic disease with median overall survival of ~1 year [1]. Currently, tebentafusp, a bispecific protein consisting of a soluble anti-CD3 effector fused to an HLA-A*02:01-restricted T-cell receptor that recognises gp100, is the only treatment that improves overall survival in patients with metastatic UM [2]. Tebentafusp therapy is limited to the 50% of patients who are HLA-A*02:01-positive and although the objective response rate was only 9% in patients treated with tebentafusp, the 1-year overall survival was significantly improved from 59% in control-treated patients to 73% in the tebentafusp-treated cohort (hazard ratio for death, 0.51; CI: 0.37–0.71; *P* > 0.001) [2].

The mutation profile of UM has also led to clinical trials with selective protein kinase C (PKC) inhibitors. PKC is activated in almost all UM via hotspot-activating mutations in *GNAQ*, *GNA11*, *CYSLTR2* or *PLCB4* genes. These oncogenic mutations promote the activation of multiple pathways, including PKC, the mitogen-activated protein kinase (MAPK) and phosphoinositide 3-kinase (PI3K)/AKT cascades [3–5]. The PKC inhibitors, AEB071 and IDE196 (darovasertib, previously known as LXS196), have shown limited clinical activity, with only 4/153 (3%) and 6/66 (9%) metastatic UM patients achieving partial responses to AEB071 and IDE196 treatment, respectively [6, 7]. Importantly, PKC inhibition suppressed the phosphorylation of the downstream effector protein MARCKS in on-treatment patient biopsies and UM cell lines regardless of PKC inhibitor response [6–8]. The PKC inhibitor-induced suppression of MARCKS phosphorylation in UM cell lines correlated with the degree of MAPK inhibition but was not associated with PI3K/AKT pathway inhibition [8]. These data suggest that the PI3K/AKT survival network may be independent of PKC activity and unresponsive to PKC inhibitor therapy in UM.

In this study, we investigated the regulation and contribution of MAPK and PI3K/AKT signalling in UM responses to PKC inhibition. Transcriptomic analyses of tumour biopsies from UM patients treated with the PKC inhibitor IDE196 reveal that the PI3K/AKT pathway is active in UM and is not inhibited by PKC inhibition. Further, the independent activation of the MAPK and PI3K/AKT signalling cascades was sufficient to overcome PKC inhibitor-mediated UM proliferative arrest and cell death in pre-clinical UM models. Importantly, the concurrent inhibition of PKC and PI3K/AKT signalling synergistically induced potent UM cell death in a panel of GNAQ/GNA11-mutant UM cell lines. Our study confirms PKC-independent activation of survival signalling cascades in UM and indicates that combinatorial treatments targeting independent signalling nodes may provide additional benefits in improving treatment response.

## Materials and methods

### Patients and clinical samples

This study included 11 patients with metastatic UM treated with the PKC inhibitor IDE196, alone or in combination with the human double minute 2 (HDM2) inhibitor HDM201 at Westmead Hospital, Sydney, Australia, as part of an experimental dose-escalation phase I clinical trial (NCT02601378) between November 2016 to August 2018 [7]. Patient demographics, performance status, UM mutation status, pre-treatment LDH levels (units/litre; U/L), treatment and biopsy details are provided in Supplementary Table S1. Investigator-determined objective responses were assessed radiologically with computed tomography (CT) scans at two monthly intervals using Response Evaluation Criteria in Solid Tumours (RECIST) 1.1 criteria. Responders were defined as patients who had partial response (PR) or stable disease (SD) for ≥6 months. Non-responders were defined as patients who had SD < 6 months or progressive disease. Clinical progression was defined by primary clinician’s assessment of disease progression in patients without re-staging imaging and were classified as progressive disease. Written consent was obtained from all patients under approved Human Research Ethics Committee protocols from Royal Prince Alfred Hospital (Protocol X15-0454 and HREC/11/RPAH/444). Fresh tumour biopsies were collected pre-treatment (PRE; median 7 days prior to treatment initiation), and early during treatment (EDT; median 26 days, range 12–29 days after treatment initiation; Supplementary Table S1) for RNA sequencing. Core biopsy samples were reviewed for tumour cell content and suitability for RNA sequencing by independent anatomical pathologists.

### RNA isolation, sequencing and analysis

Total RNA was isolated from fresh frozen core tissue sections using the AllPrep DNA/RNA/miRNA Universal kit (Qiagen, Hilden, Germany). cDNA synthesis and library construction were performed using the TruSeq RNA Library Prep Kit (Illumina) and paired-end 150 bp sequencing, with each sample yielding 40–50 million reads. Sequencing was performed on the Illumina HiSeq 2500 or NovaSeq S4 platforms at the Australian Genome Research Facility.

RNA data processing was performed as described previously [9]. Absolute signature enrichment scores using filtered (counts ≥3 in at least two samples) FPKM values were determined using ssGSEA [10] (version 10.1.0) provided by GenePattern (https://cloud.genepattern.org/) with the Hallmark gene sets plus the YAP_UP geneset from the C6 oncogenic signature gene sets (Molecular Signature Database version 2023.1) and MAPK signatures derived from [11, 12]. The correlation between ssGSEA enrichment scores was calculated using the Pearson correlation coefficient in the nearest neighbour algorithm with 1000 permutations within the Morpheus web-based tool (https://software.broadinstitute.org/morpheus/).

### Cell culture and molecular inhibitors

UM cell line sources were previously described [8]. UM cell lines were authenticated by STR profiling using the StemElite ID system from Promega, and all cells tested negative for mycoplasma (MycoAlert Mycoplasma Detection Kit, Lonza, Basel). All cell lines were maintained in Roswell Park Memorial Institute-1640 media (Gibco, Thermo Fisher Scientific, Waltham, MA, USA) supplemented with 10% or 20% foetal bovine serum (FBS; Sigma-Aldrich, Germany), 20 mM HEPES (Sigma-Aldrich), and 4 mM l-glutamine (Sigma-Aldrich) at 37 °C in 5% CO_2_. UM cells were treated with indicated concentrations of the PKC inhibitor IDE196 (Chemgood, VA, USA, C-1368), the MEK inhibitor trametinib (SelleckChem, S2673), and the PI3K/mTOR inhibitor BEZ235 (SelleckChem, S1009), all prepared in dimethyl sulfoxide (DMSO; Sigma-Aldrich). Effective drug concentrations were selected based on viability assays performed on a panel of uveal melanoma cell lines (Supplementary Fig. S1) and a review of relevant literature [8, 13–16].

### Lentiviral transduction

Lentiviral constructs encoding AKT1^E17K^ and MEK1^E203K^ in the *plenti6.3/T0/V5-DEST* vector (Thermo Fisher Scientific, MA, USA) were prepared in HEK293T cells, as previously described [17]. An empty vector was used as a control. Plasmid constructs were mixed with polybrene (Sigma-Aldrich) at 8 µg/mL and added to UM cell lines. Transduced cells were selected using Blasticidin (Gibco) at 4 µg/mL for 2–3 weeks before experiments were performed.

### Crystal violet clonogenic assay

UM cell lines OMM1.3 and Mel270 were seeded in duplicates in six-well plates (Costar, NY, USA) at 0.8–4.0 × 10^4^ cells per well and allowed to adhere overnight before treatment with 0.05% DMSO (control), 1 µM and 5 µM of IDE196. Colony formation was monitored over a 7 to 14-day period and drug media changed twice a week. Once control-treated cells neared 100% confluency, cells were washed with 1× phosphate buffer saline (PBS; Gibco), fixed with ice-cold 100% methanol, and stained with 0.1% crystal violet (Sigma-Aldrich) solution prepared in MilliQ water. The plates were imaged on ChemiDoc MP imaging system (Bio-Rad, CA, USA), and colony areas were quantified in ImageJ version 1.53a software [18] using the colony area plug-in [19]. Four biological replicates were performed per cell line.

### Flow cytometry cell cycle and apoptosis assay

UM cells were seeded in six-well plates (Costar) at 1 × 10^5^ cells per well and treated the next day with DMSO (control; final concentrations 0.04–0.08%), 10 nM Trametinib, 2 µM BEZ235, or 5 µM IDE196 for 72 h. For synergy studies, UM cells were treated with increasing doses of IDE196 (0, 0.5, 1, 2, 4 and 8 µM) and BEZ235 (0, 0.125, 0.25, 0.5, 1 and 2 µM) at a fixed 4:1 ratio for 72 h. Floating and adhered cells were collected after treatment and stained with propidium iodide, as previously described [20] and acquired on the BD LSR Fortessa X20 flow cytometer (BD Biosciences). Cell cycle and sub-G1 (cell death) analyses were performed using BD FACSDiva Software version 8.0.2 (BD Biosciences, NJ, USA) and ModFIT LT 5.0 (Verity Software House, ME, USA) to quantify DNA contents. The percentage of S-phase inhibition was calculated as [(percentage of S-phase in the control-treated cells – percentage of S-phase in drug-treated cells)/(percentage of S-phase in the control-treated cells) × 100]. Change in % sub-G1 was calculated relative to the control-treated cells (% sub-G1 in drug-treated cells – % sub-G1 in control-treated cells). Data were derived from three to four independent biological replicates.

Apoptosis in lentivirally transduced OMM1.3 and Mel270 cells was measured using Annexin V staining. Transduced and treated cells were fixed with 4% paraformaldehyde using the BD Cytofix fixation buffer (BD Biosciences), stained with PE/Dazzle 594 Annexin V (BioLegend, CA, USA) 72 h after treatment, in accordance with the suppliers’ instructions, and acquired on the BD LSR Fortessa X20 flow cytometer (BD Biosciences). Annexin V positivity was quantified using the FlowJo software v8 (BD Biosciences).

### Drug synergy analysis

Synergy analyses were based on sub-G1 data derived from the cell cycle analysis. The fraction affected values were calculated as an average sub-G1 fraction of drug-treated cells – average sub-G1 from control-treated cells, from three to four independent experiments per cell line. Synergistic activity was assessed using the Chou-Talalay method [21] and determined from CalcuSyn version 2.11 (ComboSyn Inc., Paramus, NJ, USA).

### Western blot analysis

UM cells were seeded in T75 flasks (Falcon, NY, USA) at 1.0 × 10^6^ cells per flask and treated the next day with DMSO (control), Trametinib, BEZ235, IDE196, or IDE196 ± BEZ235 for 24 h at the indicated concentrations. Cells were pelleted and lysed with RIPA lysis buffer supplemented with complete protease inhibitor cocktail (Roche, Basel, Switzerland) and PhosSTOP (Sigma-Aldrich). Protein lysates (20 µg) were loaded onto 8–10% resolving SDS-PAGE and transferred to Immobilon-FL (Sigma-Aldrich) PVDF membranes. Membranes were incubated at 4 °C overnight in primary antibodies diluted in Intercept Blocking Buffer (TBS) (Li-Cor, Lincoln, NE, USA) or Odyssey Blocking Buffer (Li-Cor) with Tween 20 (0.05%), as follows: total MARCKS (1:1000, 2C2, WH0004082M6, Sigma-Aldrich), phosphorylated MARCKS (pMARCKS; Ser^152/156^, 1:1000, 2741 S, Cell Signaling Technology, Danvers, MA, USA), DUSP6 (1:250 or 1:1000, EPR129Y, ab76310, Abcam, Cambridge, UK), total AKT (1:1000, 40D4, 2920S, Cell Signaling Technology), phosphorylated AKT (pAKT; Ser^473^, 1:100, D9E, 4060S, Cell Signaling Technology), pAKT (Ser^473^, 1:500, 736E11, 3787, Cell Signaling Technology), total ribosomal S6 (1:500, 54D2, 2317 S, Cell Signaling Technology), phosphorylated ribosomal S6 (pS6; Ser^235/236^, 1:1000, 2F9, 4856S, Cell Signaling Technology), total YAP (1:500, 1A12, 12395S, Cell Signaling Technology), phosphorylated YAP (pYAP; Ser^127^, 1:2000, 4911, Cell Signaling Technology), total ERK (1:2 000, 137F5, 4695S, Cell Signaling Technology), phosphorylated ERK (pERK; Tyr^204^, 1:250, E-4, SC-7383, Santa Cruz, Dallas, TX, USA), and MEK1/2 (1:500, L38C12, 4694S, Cell Signaling Technology). Membranes were washed with tris-buffered saline with 0.05% Tween 20 (TTBS) and incubated for one hour in secondary antibodies IRDye® 800CW Rabbit anti-Mouse, IRDye® 800CW Mouse anti-Rabbit, IRDye® 680LT Rabbit anti-Mouse or IRDye® 680LT Mouse anti-Rabbit (all at 1:20,000, Li-Cor), diluted 1:1 in Intercept Blocking Buffer (Li-Cor) or Odyssey Blocking Buffer (Li-Cor) with TTBS. Bands were imaged using the Odyssey imaging system. DUSP6 signals were normalised using REVERT total protein stain (Li-Cor), and phosphorylated proteins were normalised against their respective total proteins. Normalised protein data were log_2_ transformed, and independent experiments were averaged. Data were derived from three to four independent biological replicates.

### Statistical analysis

All statistical analyses were performed using the GraphPad Prism 9.4 software (GraphPad, CA, USA) and statistical significance between groups was determined using one-way ANOVA with the Geisser–Greenhouse correction and Tukey’s or Sidak’s multiple comparison test, with individual variances computed for each comparison. Data was derived from at least three biological replicates, unless otherwise noted, and shown as mean ± SD. The D’Agostino and Pearson and Shapiro–Wilk test (suitable for smaller sample sizes) was used to assess data normality.

## Results

### Patient and tumour characteristics

Transcriptome analysis was performed on patient-matched PRE and EDT core biopsies of UM metastases derived from 11 patients treated with the PKC inhibitor-based therapy as part of a phase I clinical trial (NCT02601378). The median age was 67 years, and the majority of patients were female (6/11; 55%), with choroidal primary UM diagnosed in 9/11 (82%) patients. All patients had an established UM driver mutation with *GNAQ* activating mutations identified in 8/11 (73%) UM biopsies. The median progression-free survival (PFS) was 3.8 months (Supplementary Table S1). Patients were treated with PKC inhibitor IDE196 monotherapy (*n* = 5) or with the combination of IDE196 with the HDM2 inhibitor, HDM201 (*n* = 6). Due to the small numbers of available UM biopsies, we combined the PKC inhibitor monotherapy and PKC plus HDM2 inhibitor-treated patients. None of these patients had PR as per RECIST 1.1 to PKC inhibitor-based therapy; two patients (18%) were considered responders with SD ≥6 months, and nine patients (82%) were considered non-responders; 6/9 (67%) patients had SD <6 months and 3/9 (33%) patients had progressive disease.

### PKC inhibitor treatment suppresses MAPK signalling and UM proliferation in vivo

**Fig. 1:**
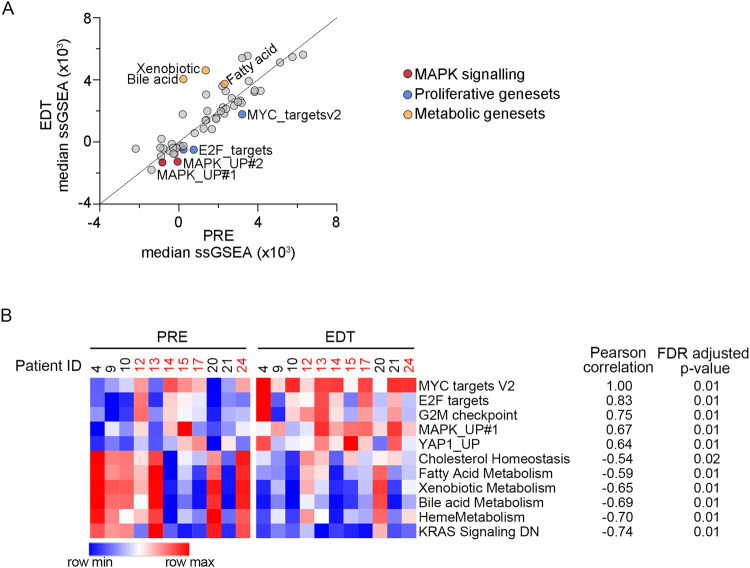
PKC inhibitor treatment suppresses MAPK signalling in vivo. **A** Median ssGSEA scores for the Hallmark and MAPK gene sets in 11 UM PRE and EDT tumour biopsies treated with the PKC inhibitor IDE196 monotherapy (*n* = 5) or with combination IDE196 and the HDM2 inhibitor, HDM201 (*n* = 6). MAPK signalling gene sets are shown in red, proliferative gene sets in blue and metabolic gene sets in yellow. **B** Correlation of the proliferative Hallmark_MYC_targets_V2 targets signature (ssGSEA scores) with other gene sets in tumour biopsies of UM patients treated with IDE196 monotherapy (*n* = 5, black) or with combination IDE196 and the HDM2 inhibitor, HDM201 (*n* = 6, red). The correlation was calculated using Pearson’s correlation, false discovery rate (FDR)-adjusted *P* values shown.

We first sought to examine whether UM transcriptome signalling changes were occurring early during therapy (EDT) in the 11 patients treated with the PKC inhibitor-based treatment. Single sample gene set enrichment (ssGSEA) scores [10], which represent the activity level of well-defined processes, were derived from the transcriptome data of each UM biopsy (Supplementary Table S2). Comparison of ssGSEA scores confirmed that the activity of most processes was comparable in baseline (PRE) and EDT UM biopsies. However, gene sets associated with proliferation and MAPK signalling were consistently diminished post-PKC inhibitor treatment while metabolic signatures, including bile-acid metabolism, xenobiotic metabolism and fatty acid metabolism were induced in response to PKC inhibitor therapy (Fig. 1A). Interestingly, the proliferative gene set Hallmark_MYC_targets_V2 positively correlated with other proliferative and MAPK gene sets and negatively correlated with metabolic signatures (Fig. 1B and Supplementary Table S3). These data validate previous PKC inhibitor analyses in UM cell lines [8], confirming that although PKC inhibition suppressed MAPK signalling and tumour cell proliferation, this was not sufficient to produce meaningful clinical benefits in patients with advanced UM.

### Selective inhibition of MAPK or PI3K/AKT signalling has minimal impact on UM cell survival

**Fig. 2:**
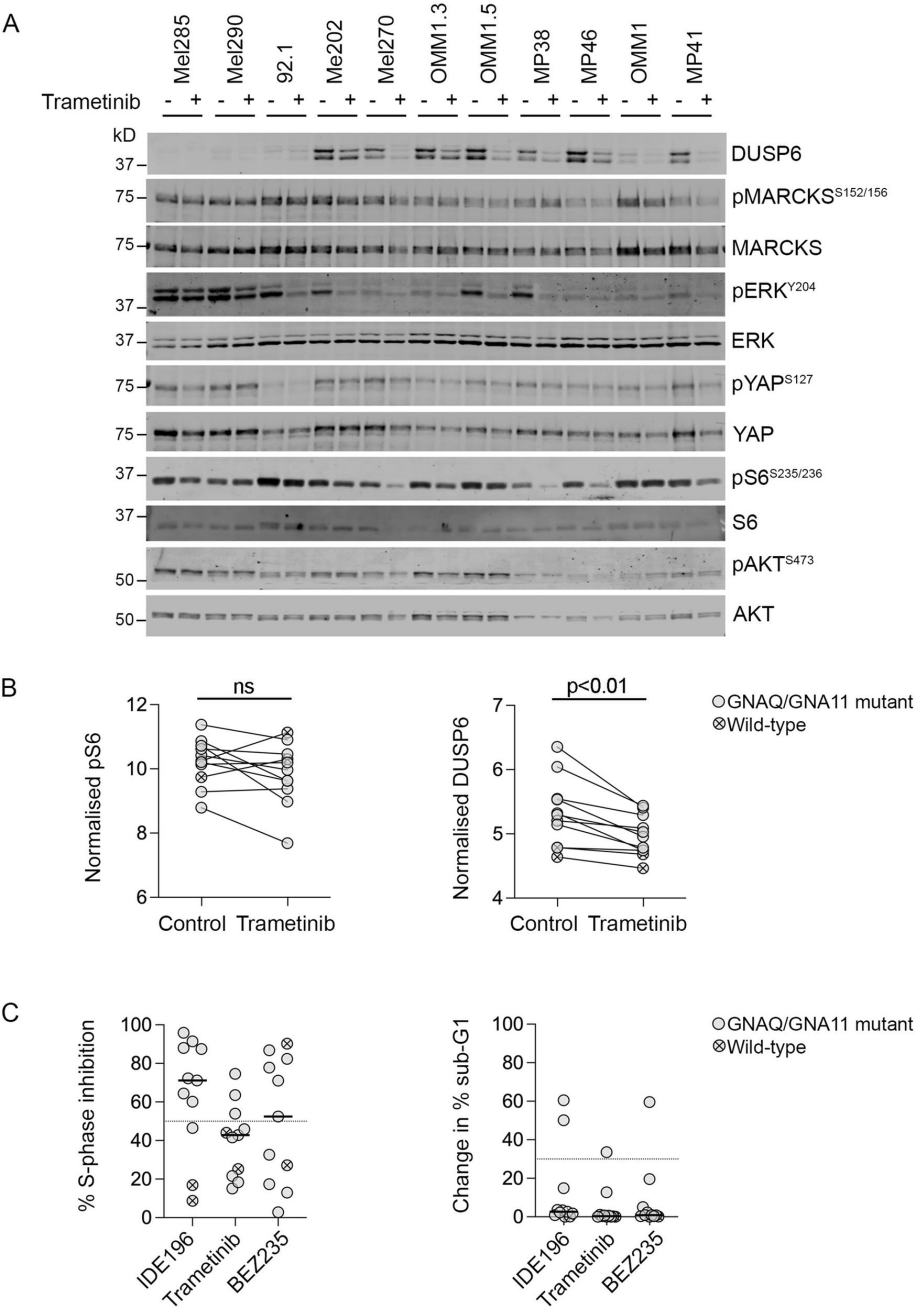
Inhibition of MAPK signalling with Trametinib in UM cell lines. **A** Accumulation of MAPK, PI3K/AKT, YAP and PKC signalling effectors including total and phosphorylated DUSP6, MARCKS, ERK, YAP, S6 and AKT 24 h after treatment with vehicle control (−) or 10 nM Trametinib (+). Western blot analyses were performed using three independent biological replicates (*n* = 3). kD kilodalton. REVERT total protein loading stain is shown in Supplementary Fig. S4. **B** Expression levels of pS6 and DUSP6 (normalised to total S6 or REVERT staining, respectively) in vehicle control or 10 nM Trametinib-treated *GNAQ/GNA11*-mutant (solid dot) or wild-type (crossed dot) UM cell lines. Data derived from three independent biological experiments (*n* = 3), and *P* values were calculated using paired *t* tests. ns not significant. **C** Percentage of cells undergoing S-phase inhibition (dotted line set at 50% S-phase inhibition) and change in % sub-G1 (dotted line set at 30% sub-G1) in *GNAQ/GNA11*-mutant (solid circle) or wild-type (crossed circle) UM cell lines treated with 5 µM IDE196 (data derived from [8]), 10 nM Trametinib or 2 µM BEZ235. Data derived from three independent biological experiments (*n* = 3).

To expand the analyses of MAPK signalling and its relationship with PKC inhibitor sensitivity in UM, we screened a panel of 11 UM cell lines with available PKC inhibitor response data (Supplementary Table S4). Treatment with the allosteric MEK1/2 inhibitor trametinib decreased the accumulation of MAPK downstream targets DUSP6 and phosphorylated ribosomal protein S6 (pS6) in 7/9 *GNAQ/GNA11*-mutant and 0/2 wild-type UM cell lines (Fig. 2A, B). The inhibition of MAPK signalling was usually not sufficient to promote significant proliferative arrest (3/11 cell lines with >50% S-phase inhibition) or cell death with only the Mel270 UM cell line showing >30% sub-G1 change with Trametinib treatment (Fig. 2C).

**Fig. 3:**
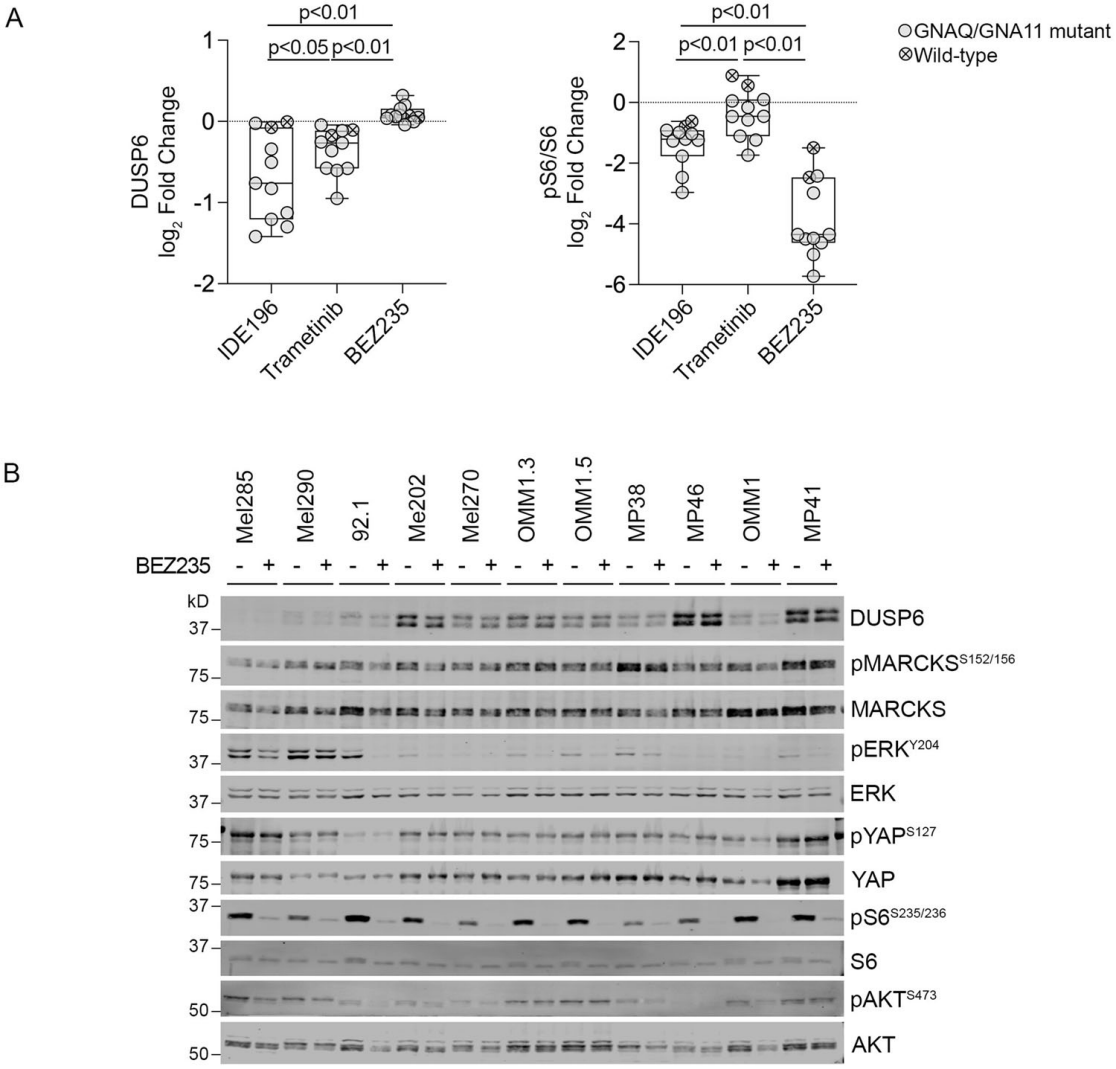
Impact of PKC, PI3K/AKT and MAPK inhibition in UM cell lines. **A** Fold change in DUSP6 and pS6 expression (normalised log_2_ protein expression in drug-treated – normalised log_2_ protein expression in control-treated cells) in *GNAQ/GNA11*-mutant (solid circle) and wild-type (crossed circle) UM cell lines treated with 5 µM IDE196, 10 nM Trametinib, or 2 µM BEZ235. Data compared using one-way ANOVA with the Geisser–Greenhouse correction and Tukey’s multiple comparison test, adjusted *P* values are shown. Data derived from three independent biological experiments (*n* = 3, mean ± SD). **B** Accumulation of MAPK, PI3K, YAP and PKC signalling effectors, including total and phosphorylated DUSP6, MARCKS, ERK, YAP, S6 and AKT 24 h after treatment with vehicle control (−) or 2 µM BEZ235 (+). Western blot analyses were performed using three independent biological replicates (*n* = 3). kD kilodalton. REVERT total protein loading stain is shown in Supplementary Fig. S5.

In this panel of UM cell models, PKC inhibition was more effective than MAPK inhibition in promoting UM cell cycle arrest (8/11 cell lines with >50% S-phase inhibition) and/or cell death (2/11 cell lines with >30% sub-G1 change) (Supplementary Table S4), and this was presumably due to PKC inhibitor-mediated suppression of MAPK-independent networks. In fact, we noted that the degree of pS6 inhibition was more pronounced in response to PKC inhibition with IDE196 treatment compared to MAPK inhibition with trametinib treatment (Fig. 3A). It is well established that S6 phosphorylation is induced by multiple pathways, including the MAPK and PI3K/AKT signalling cascades [22]. Indeed, inhibition of the PI3K/AKT signalling cascade with the dual PI3K and mTOR inhibitor BEZ235 potently suppressed the phosphorylation of S6 in all 11 UM cell lines (including the two wild-type UM cell lines; Fig. 3A, B). It is worth noting that PKC inhibition did not achieve the level of pS6 inhibition seen with BEZ235 treatment (Fig. 3A), and this aligns with our data showing that the Hallmark_MTORC1_Signalling and Hallmark_PI3K_AKT_MTOR_Signalling showed minimal downregulation in UM tissue specimens early during treatment with PKC inhibitor (Supplementary Table S2). The inhibition of PI3K/mTOR signalling with BEZ235 treatment resulted in proliferative inhibition (>50% S-phase inhibition) in 5/7 GNAQ/GNA11-mutant UM and in the wild-type Mel290 cell lines (Fig. 2C). This translated to minimal cell death, however, and only the OMM1 cell line showed substantial cell death (60% sub-G1 change) in response to 2 µM BEZ235 (Fig. 2C).

### MAPK and PI3K/AKT pathways differentially contribute to PKC inhibitor responses

Our data confirm that MAPK and PI3K/AKT pathways are active in UM, but only the MAPK pathway is dependent on PKC activity. Thus, we examined whether these pathways regulate PKC inhibitor sensitivity in UM. The MAPK and PI3K/AKT pathways were each independently activated in UM by introducing the gain-of-function MEK1^E203K^ and AKT1^E17K^ mutations, respectively, into the *GNAQ*-mutant OMM1.3 and Mel270 UM cell models. These cell lines were chosen given their differing responses to PKC inhibitor monotherapy (Supplementary Table S4); OMM1.3 cells were more resistant to 1 µM IDE196, with only 8.4% increase in the sub-G1 population following treatment compared to an increase of 65% sub-G1 population in Mel270 cells treated with the same dose (Supplementary Table S4).

**Fig. 4:**
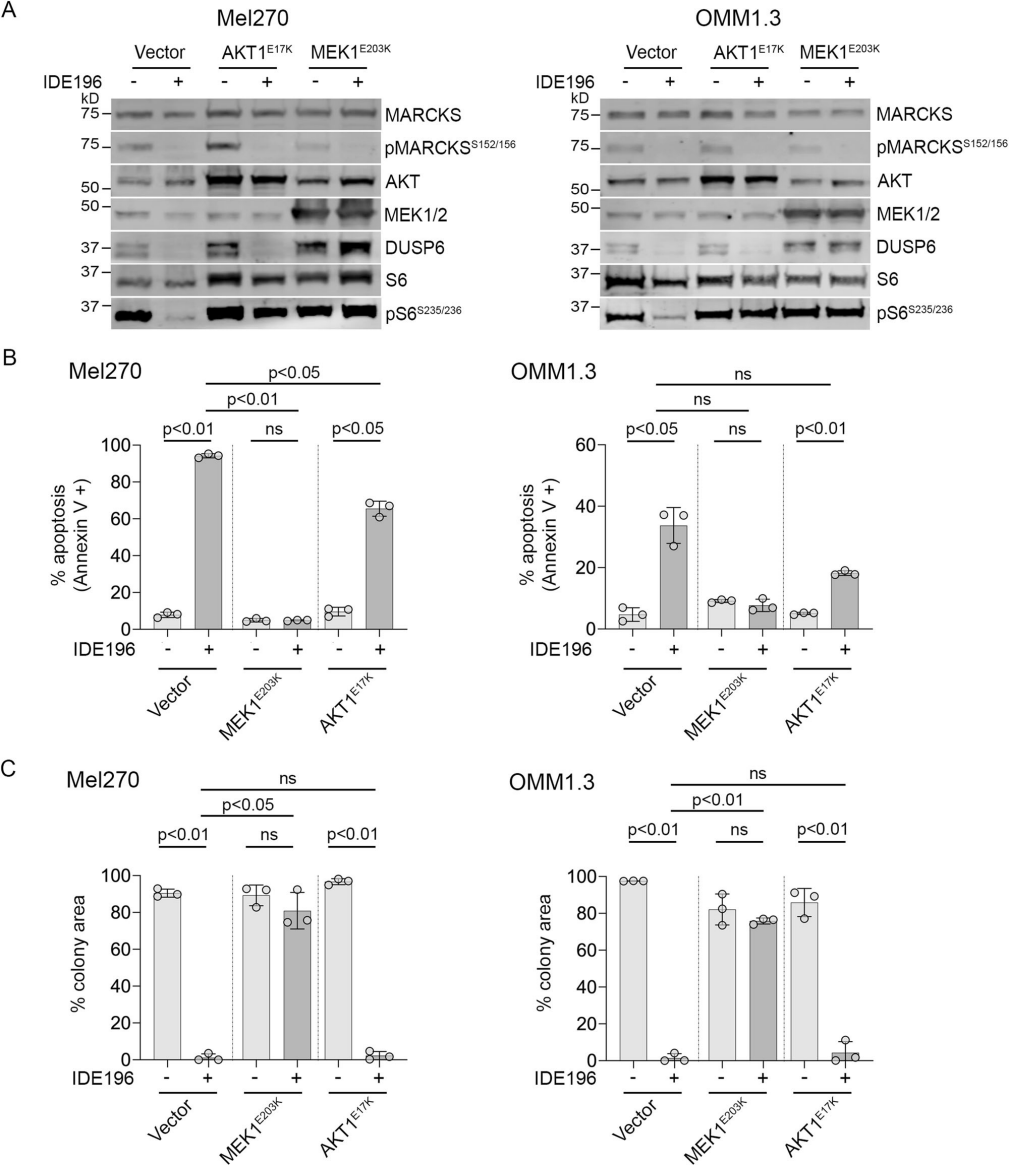
Oncogenic activation of MAPK and PI3K/AKT signalling circumvents PKC inhibition in UM. **A** Accumulation of MAPK and PI3K/AKT signalling effector proteins in Mel270 and OMM1.3 UM cell lines transduced with lentiviral expression constructs AKT1^E17K^, MEK1^E203K^, or empty vector control 24 h post treatment with 1 µM IDE196 (+) or vehicle control (−). Western blot analyses were performed twice using independent biological replicates (*n* = 2) derived from one transduction. kD kilodalton. REVERT total protein loading stains are shown in Supplementary Fig. S6. **B** Percentage of apoptotic Mel270 and OMM1.3 cells (Annexin V + ) expressing ectopic control vector, AKT1^E17K^ or MEK1^E203K^ 72 h after treatment with 1 µM IDE196. Data represents mean ± SD of three biological replicates (*n* = 3) derived from one transduction and compared using one-way ANOVA with the Geisser–Greenhouse correction and Sidak’s multiple comparison test, adjusted *P* values are shown. **C** Percentage of colony area occupied by control, AKT1^E17K^ or MEK1^E203K^-expressing Mel270 and OMM1.3 cells 7–10 days after exposure to 1 µM IDE196. Data represents mean ± SD of three biological replicates (*n* = 3) derived from one transduction and compared using one-way ANOVA with the Geisser–Greenhouse correction and Sidak’s multiple comparison test, adjusted *P* values are shown. ns not significant.

The ectopic expression of the MEK1^E203K^ and AKT1^E17K^ transgenes was confirmed by immunoblotting (Fig. 4A). As expected, treatment with 1 µM IDE196 for 24 h potently suppressed the phosphorylation of the PKC effector protein MARCKS in all transduced cell models, confirming the activity of the PKC inhibitor (Fig. 4A). In the presence of MEK1^E203K^, however, IDE196 did not suppress the expression of the MAPK downstream target DUSP6 nor the phosphorylation of the PI3K/AKT and MAPK co-effector protein, S6. Similarly, ectopic AKT1^E17K^ expression diminished the IDE196-mediated inhibition of pS6 (Fig. 4A). Thus, MAPK and PI3K/AKT signalling remained activated in the presence of MEK1^E203K^ and AKT1^E17K^, irrespective of PKC inhibition in the OMM1.3 and Mel270 UM cell lines.

The ectopic expression of MEK1^E203K^ or AKT1^E17K^ also diminished IDE196-induced apoptosis in both the OMM1.3 and Mel270 cell lines (Fig. 4B and Supplementary Fig. S2A). Activation of MAPK signalling was more potent than PI3K/AKT pathway activation in mediating PKC inhibitor resistance in both UM models. The MEK1^E203K^ oncogene also promoted significant outgrowth of Mel270 and OMM1.3 colonies in the presence of 1 µM IDE196 in longer-term clonogenic assays (Fig. 4C and Supplementary Fig. S2B). In contrast, ectopic AKT1^E17K^ expression resulted in the formation of small visible colonies, although these could not be accurately quantitated (Supplementary Fig. S2B). These data confirm that the MAPK pathway is a critical target of PKC inhibitor activity in UM, with the PI3K/AKT pathway contributing to the survival of UM cells in a PKC-independent manner.

### Combination of PKC and PI3K/AKT pathway inhibition leads to enhanced UM cytotoxicity

**Fig. 5:**
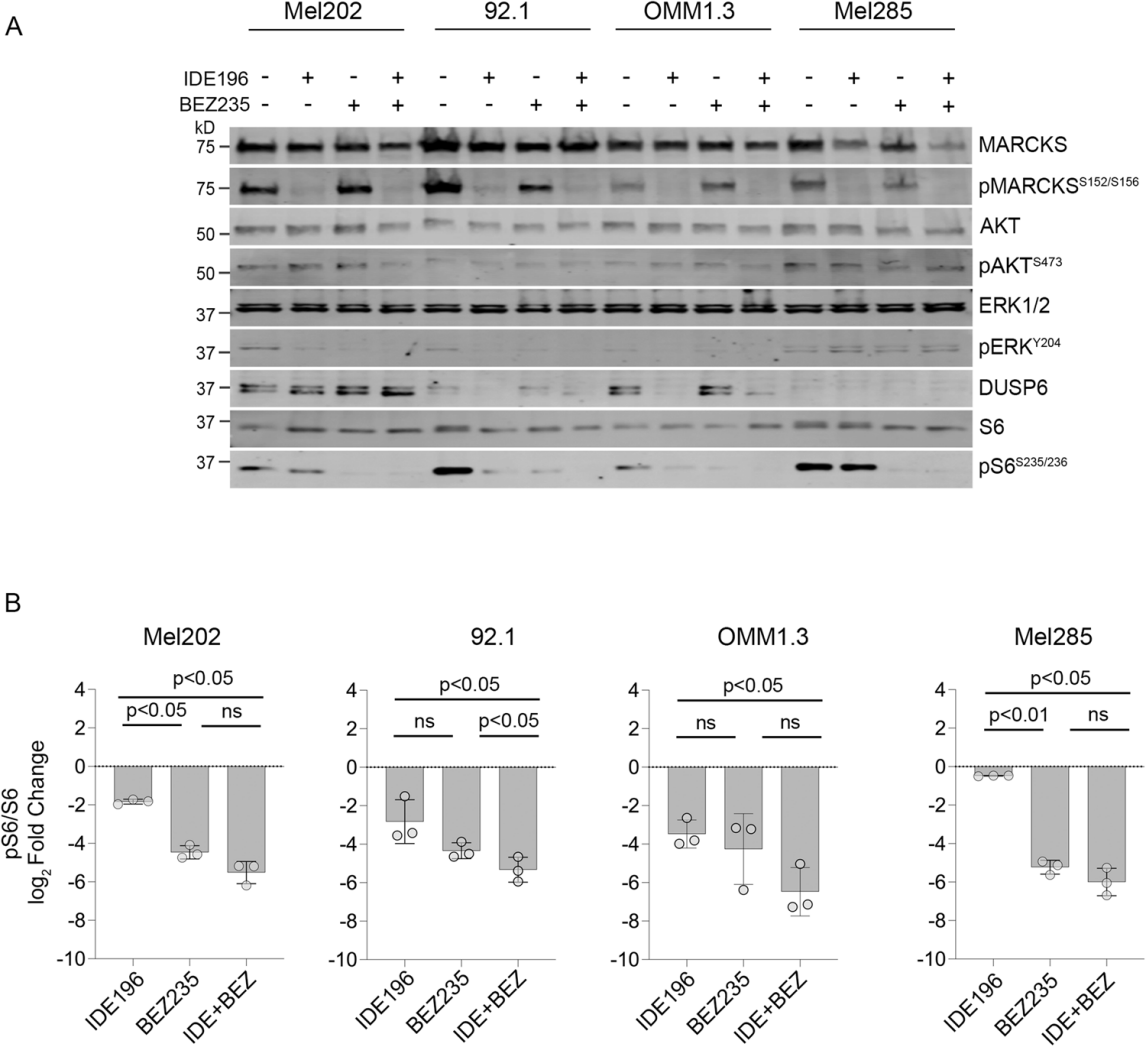
Concurrent inhibition of PKC and PI3K/AKT pathways promote synergistic cell death in UM. **A** Accumulation of MAPK and PI3K/AKT signalling effector proteins 24 h after treatment with vehicle control (−), 4 µM IDE196, 1 µM BEZ235, or combination 4 µM IDE196 and 1 µM BEZ235. kD, kilodalton, REVERT total protein stain was performed as loading control (Supplementary Fig. S7). **B** Fold change in pS6 expression (normalised log_2_ protein expression in drug-treated – normalised log_2_ protein expression in control-treated cells) in Mel202, 92.1, OMM1.3, and Mel285 UM cell lines (*n* = 3, mean ± SD). Data compared using one-way ANOVA with the Geisser–Greenhouse correction and Tukey’s multiple comparison test, adjusted *P* values are shown. kD kilodalton, ns not significant.

Our data confirm that PI3K/AKT signalling is independent of PKC activity and diminishes PKC inhibitor sensitivity in UM cell lines. To investigate whether the concurrent inhibition of the PKC and PI3K/AKT pathways is effective in inducing potent cell death, we treated seven UM cell lines (*GNAQ*-mutant Mel202, OMM1.3, and 92.1, *GNA11*-mutant MP41 and OMM1, and *GNAQ/GNA11* wild-type Mel285 and Mel290) with increasing fixed-dose combinations of IDE196 and BEZ235. Apart from OMM1, 92.1, and Mel202, all cell lines showed minimal to no cell death (change in % sub-G1 < 10%) in response to either PKC or PI3K/mTOR inhibitor monotherapy (Supplementary Table S4). When treated with the combination of IDE196 and BEZ235 at a 4:1 dose ratio, 4/7 cell lines (Mel202, 92.1, MP41, and OMM1) showed strong synergistic cell death with combination indexes (CI) of <1.0 at the median effective dose (ED_50_, Supplementary Table S5 and Supplementary Fig. S3). Further, although IDE196 and BEZ235 monotherapy promoted minimal (<10%) cell death in the OMM1.3 UM cell line, the combination of these inhibitors was more effective with 36% cell death evident at the highest doses (Supplementary Fig. S3). Neither single nor combination therapies induced cell death in the two *GNAQ/GNA11* wild-type UM cell lines Mel285 and Mel290 (Supplementary Table S4). As expected, the concurrent inhibition of PKC and PI3K/mTOR signalling induced more pronounced inhibition of S6 phosphorylation compared to either drug alone, although the degree of S6 inhibition did not reflect drug response. In particular, pS6 was similarly suppressed in the most sensitive Mel202 and the resistant Mel285 UM cell lines (Fig. 5).

## Discussion

The selective inhibition of PKC, a downstream effector of GNAQ and GNA11, provides a promising approach for the treatment of patients with UM carrying *GNAQ/GNA11* mutations. In recent phase I clinical trials, however, the PKC inhibitors AEB071 and IDE196 showed limited clinical activity, with less than 10% of metastatic UM patients achieving a partial response. The PKC inhibitors engaged with the target protein in vivo and suppressed the downstream effector MARCKS [6, 7] and MAPK signalling [8]. These data suggest that UM proliferative and survival signals may involve both PKC-dependent and -independent pathways.

In this study, the PKC dependency of MAPK signalling was confirmed with transcriptome analysis of UM biopsies collected prior to and early during IDE196 PKC inhibitor therapy. The PKC inhibitor-mediated suppression of MAPK signalling was closely correlated with diminished tumour cell proliferation, as measured by independent proliferative gene expression signatures. Importantly, the re-activation of MAPK activity, via the expression of the activating MEK1^E203K^ mutation, completely restored UM proliferation and survival in the presence of PKC inhibition. Although these data confirm the dominant role of MAPK signalling in regulating PKC inhibitor responses in UM, the selective inhibition of MAPK activity, with the allosteric MEK1/2 inhibitor Trametinib, did not recapitulate PKC inhibitor responses, and was insufficient to induce significant cell death in most UM cell lines. These data support the notion that PKC inhibition suppresses multiple pathways involved in UM survival, including but not limited to the MAPK cascade.

The PI3K/AKT signalling cascade is a central regulator of cell survival and is activated in most UM tumours. We confirmed that PI3K/AKT signalling is independent of PKC activity in UM cell lines [8], and PI3K/AKT remains active in UM biopsies derived from patients treated with the PKC inhibitor IDE196. Previous studies have shown PI3K/AKT activation in UM via the upregulation of receptor tyrosine kinases including c-MET, c-KIT, and VEGFR and via the loss of the phosphatase and tensin homologue protein (PTEN) [23–25]. Importantly, we also show that stimulation of PI3K/AKT activity, via the expression of the oncogenic AKT1^E17K^ mutation, was sufficient to partially diminish PKC inhibitor sensitivity in UM cell lines. Despite the critical role of PI3K/AKT signalling in UM, the selective inhibition of this pathway using the dual PI3K and mTOR inhibitor, BEZ235, was not sufficient to induce proliferative arrest or significant cell death in most UM cell lines.

In agreement with our pre-clinical data, clinical studies with single-agent molecular therapies in UM have been disappointing. For instance, PKC inhibitor monotherapy effectively inhibited PKC signalling, but few metastatic UM patients responded to these inhibitors and the median PFS was only 3.6 months [7, 26]. Similarly, a phase III clinical trial combining the MEK1/2 inhibitor selumetinib with dacarbazine in treatment-naïve metastatic UM patients showed a response rate of only 3% and no improvement in PFS compared with placebo and dacarbazine [27].

There is now increasing interest in combination therapies that target independent UM signalling pathways. Interim results from the phase II clinical trial (NCT03947385) evaluating the combination of PKC and c-MET inhibitors showed partial responses in 11/35 (31%) metastatic UM patients with 50% response rates in first-line UM patients [28]. We also demonstrate that whereas the sole inhibition of PKC or PI3K/AKT pathways induced minimal UM cell death, the dual inhibition of these pathways induced synergistic UM cell death in all but one *GNAQ/GNA11*-mutant UM cell line. It is worth noting that the co-inhibition of PKC and PI3K/AKT did not induce the death of *GNAQ/GNA11* wild-type UM cell lines, although this combination effectively suppressed S6 phosphorylation in these cell lines, indicating that UM cells without *GNAQ/GNA11* mutations may be less reliant on these networks.

The optimal selection and dosing of molecular therapy combination needs to be carefully considered. For instance, inhibitors of upstream regulators such as PKC may be more effective than inhibiting the mid-kinase tier of the MAPK pathway, although the suppression of multiple PKC-induced pathways may lead to more frequent and serious toxicities. For instance, a phase Ib/II clinical trial using the MEK inhibitor binimetinib combined with the PKC inhibitor AEB071 (NCT01801358) was terminated prior to phase II expansion due to toxicity. Similarly, dose reductions for adverse events were also common in metastatic UM patients receiving combination MEK and AKT inhibitors (Trametinib and GSK795) [29]. Achieving sufficient dose for adequate target suppression is also challenging, and in a phase Ib study combining the PKC inhibitor sotrastaurin with the PI3K inhibitor alpelisib, the maximum tolerated doses led to modest target inhibition, no UM patient responses, and a PFS of only 8 weeks [30]. Hence, careful design and selection of alternate molecular inhibitors or delivery methods that target PKC and the PI3K/AKT networks need to be explored to potentiate dose reduction, widen the therapeutic window, and induce effective blockade of these survival networks in UM tumours.

### Supplementary information

Supplementary Figures

Supplementary Tables

## Data Availability

The RNA sequencing data generated in this study have been submitted to the Sequence Read Archive under the accession code SUB13702225 and will be made publicly available. All other data are available within the text or Supplementary Materials.
