# Supplementary material for: PKC-independent PI3K signalling diminishes PKC inhibitor sensitivity in uveal melanoma
Source: Oncogenesis. 2024 Feb 28;13(1):9. doi: 10.1038/s41389-024-00511-8 (PMC10902289; doi:10.1038/s41389-024-00511-8)
Supplement: Supplementary file 1 — Supplementary Figures [file 41389_2024_511_MOESM1_ESM.pdf]

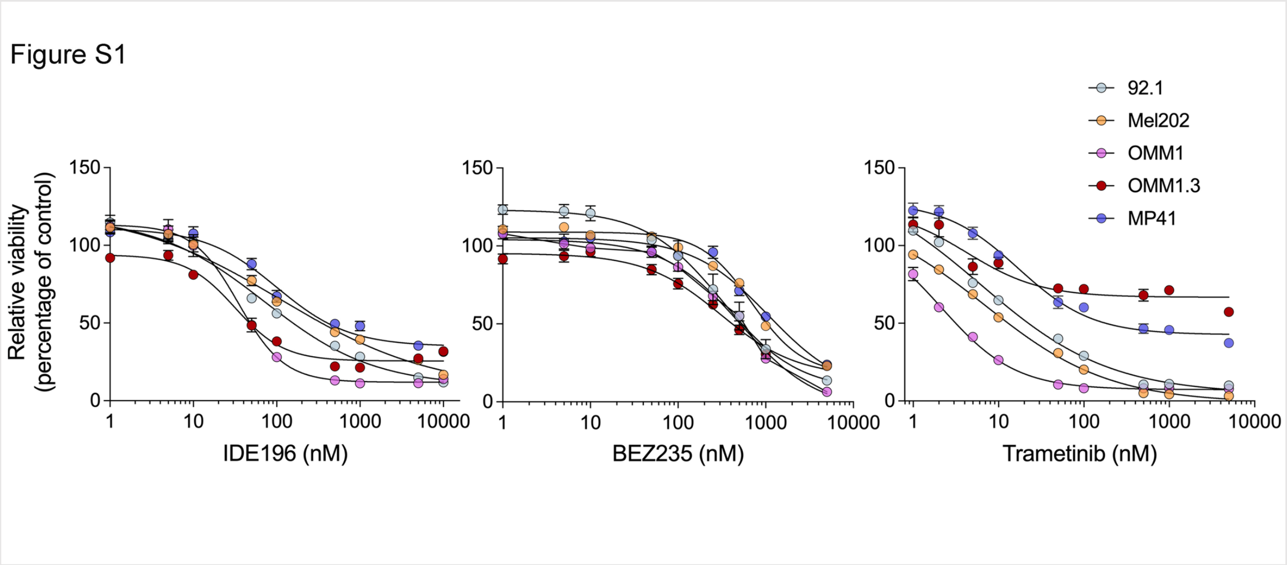

Figure S1: Drug response curves in uveal melanoma cell lines

Indicated uveal melanoma cells were treated with the indicated doses of IDE196, BEZ235 or trametinib for 72h (relative to DMSO-treated controls; mean ±SEM; n≥4)

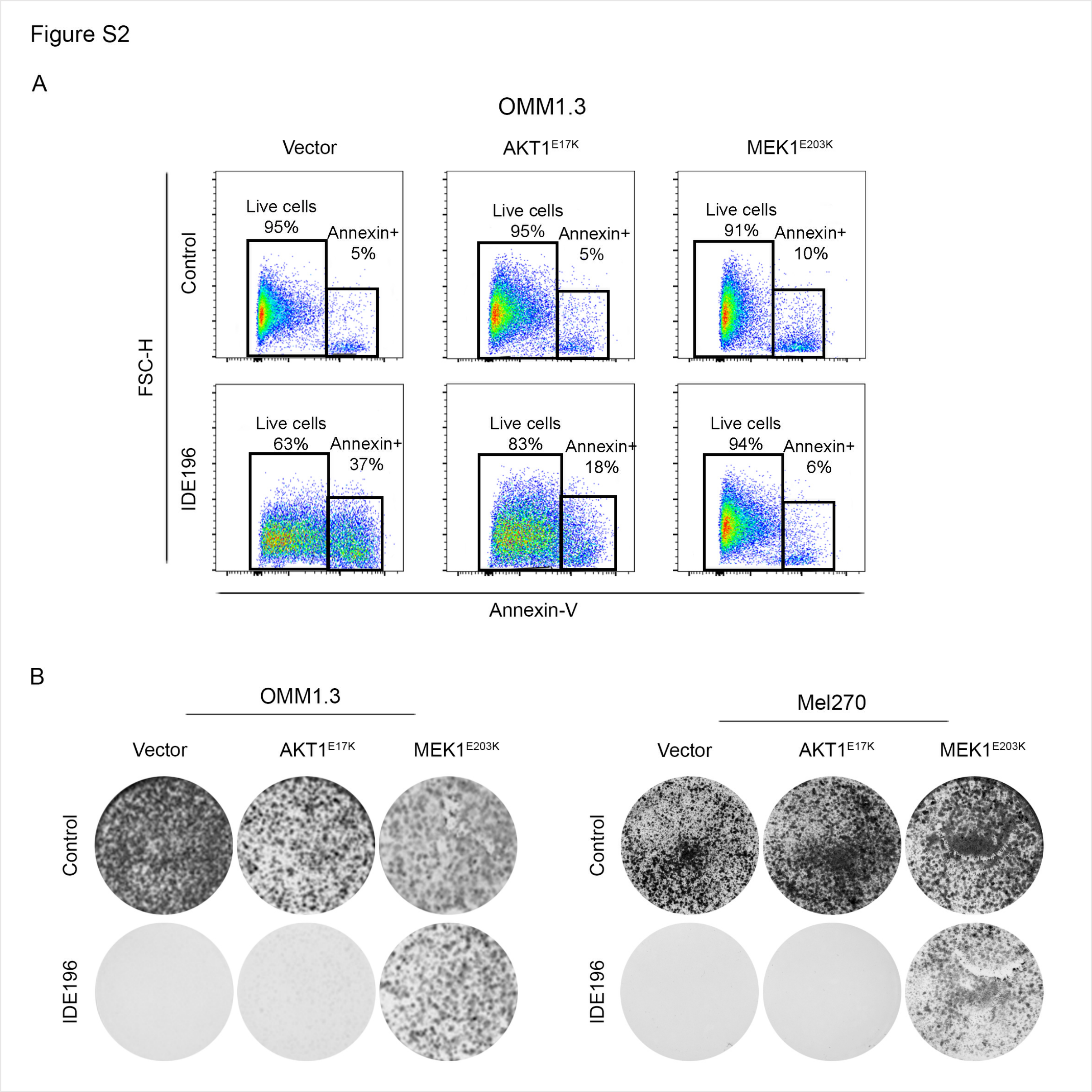

Figure S2: MAPK and PI3K reactivation overrides PKC inhibitor response in UM cells

A) Representative scatterplot showing percentage of Annexin V-positive OMM1.3 UM cells transduced with lentiviral constructs AKT1^{E17K}, MEK1^{E203K} or empty vector. Cells were stained for Annexin V, 72 h post treatment with vehicle control or 1 µM IDE196. FSC-H, forward scatter-height,

B) Representative images of colony formation in AKT1^{E17K}- and MEK1^{E203K}- transduced OMM1.3 and Mel270 UM cells. Cells were stained with 0.1% crystal violet 7 – 10 days after treatment with vehicle control or 1 µM IDE196.

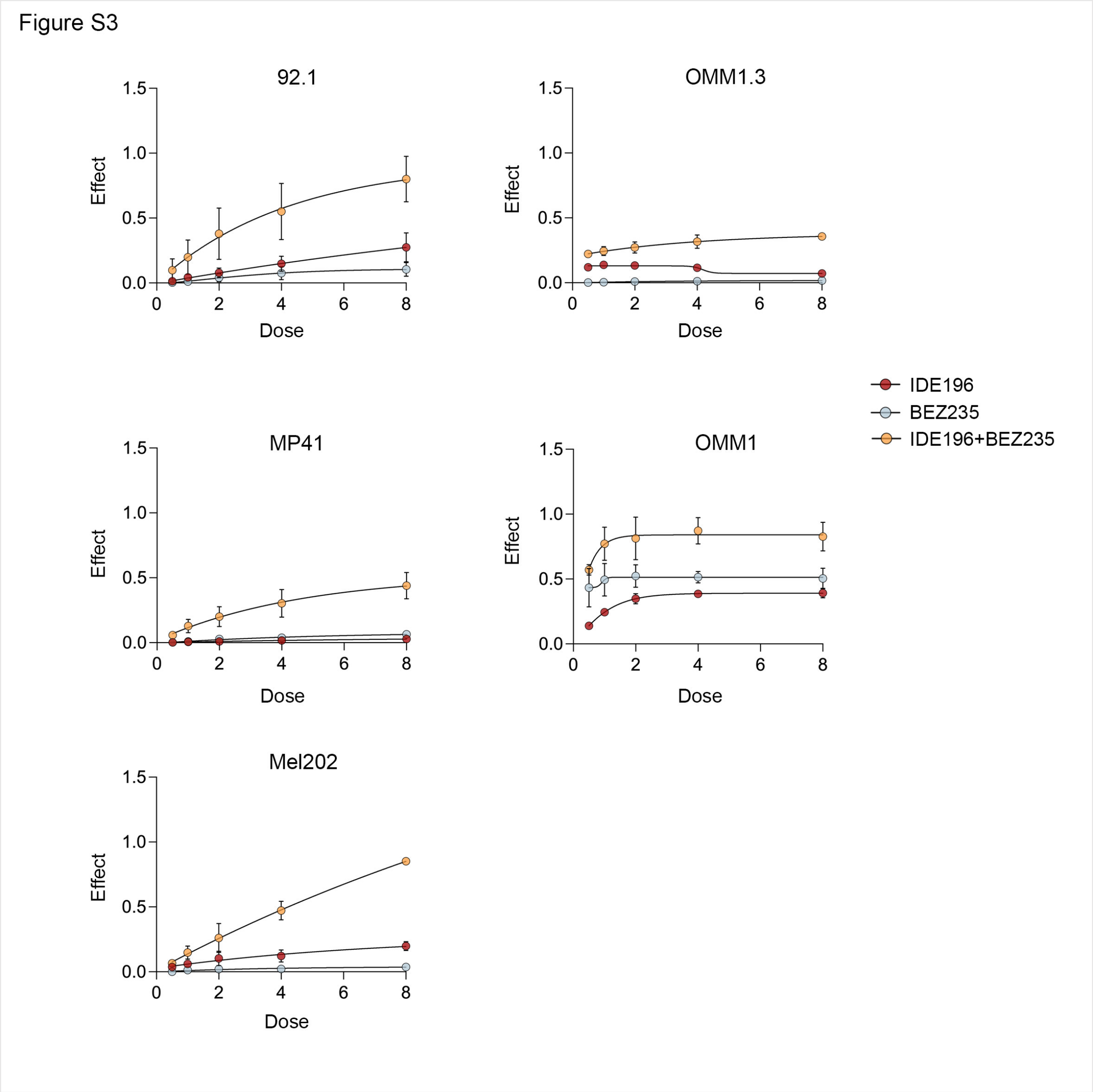

Figure S3: Dose-effect curves derived from GNAQ/GNA11-mutant UM cells treated with PKC and/or PI3K/mTOR inhibitors

Dose effect curves of five UM cell lines showing responses to concurrent IDE196 and BEZ235 inhibition. Sub-G1 data are derived from 3 – 4 independent experiments (mean ± SD). The fraction affected values (y-axis) were calculated as average sub-G1 fraction of drug-treated cells – average sub-G1 from control-treated cells, from 3 – 4 independent experiments per cell line. IDE196 dose (μM) is shown on the x-axis and BEZ235 was used at 0.25x the IDE196 dose.

Figure S4

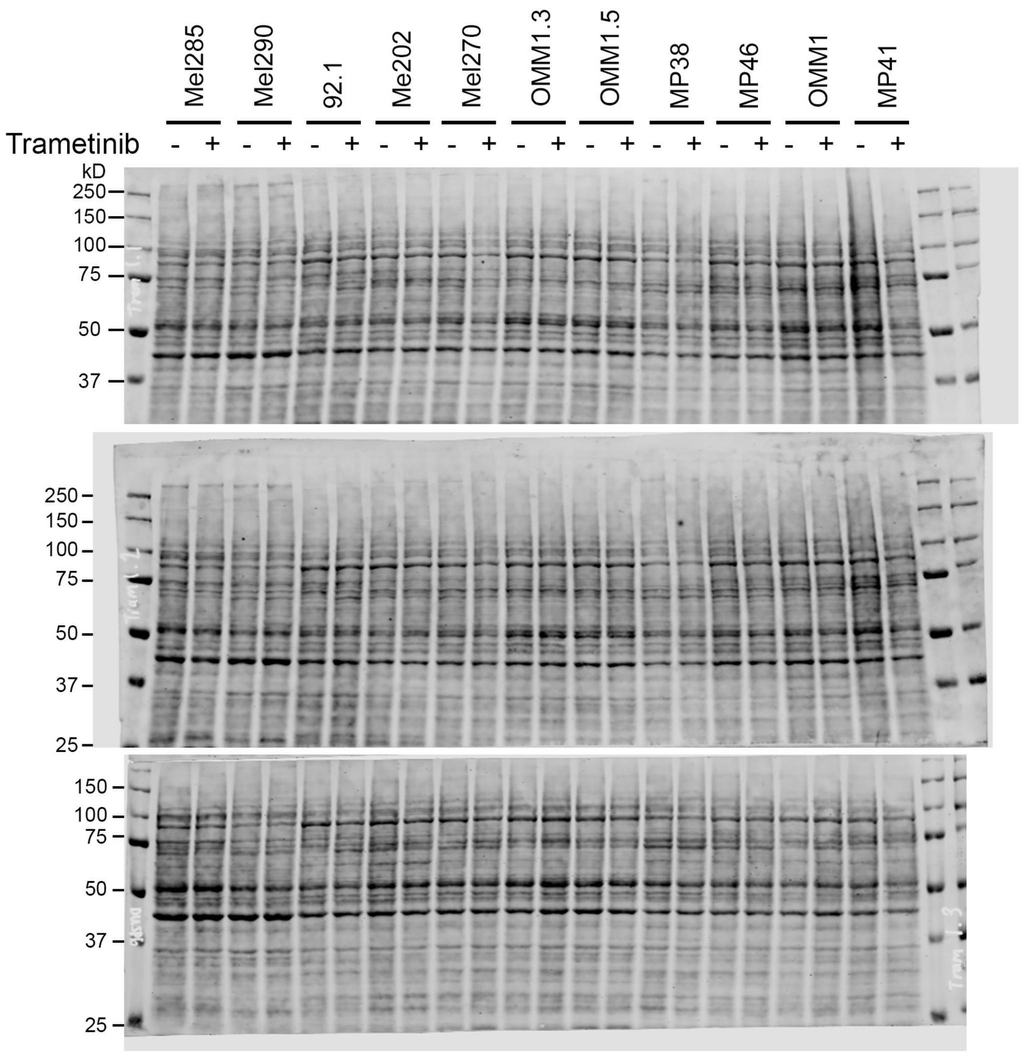

Figure S4: REVERT total protein stain

Blots showing REVERT total protein stain corresponding to Figure 2A. kD, kilodalton.

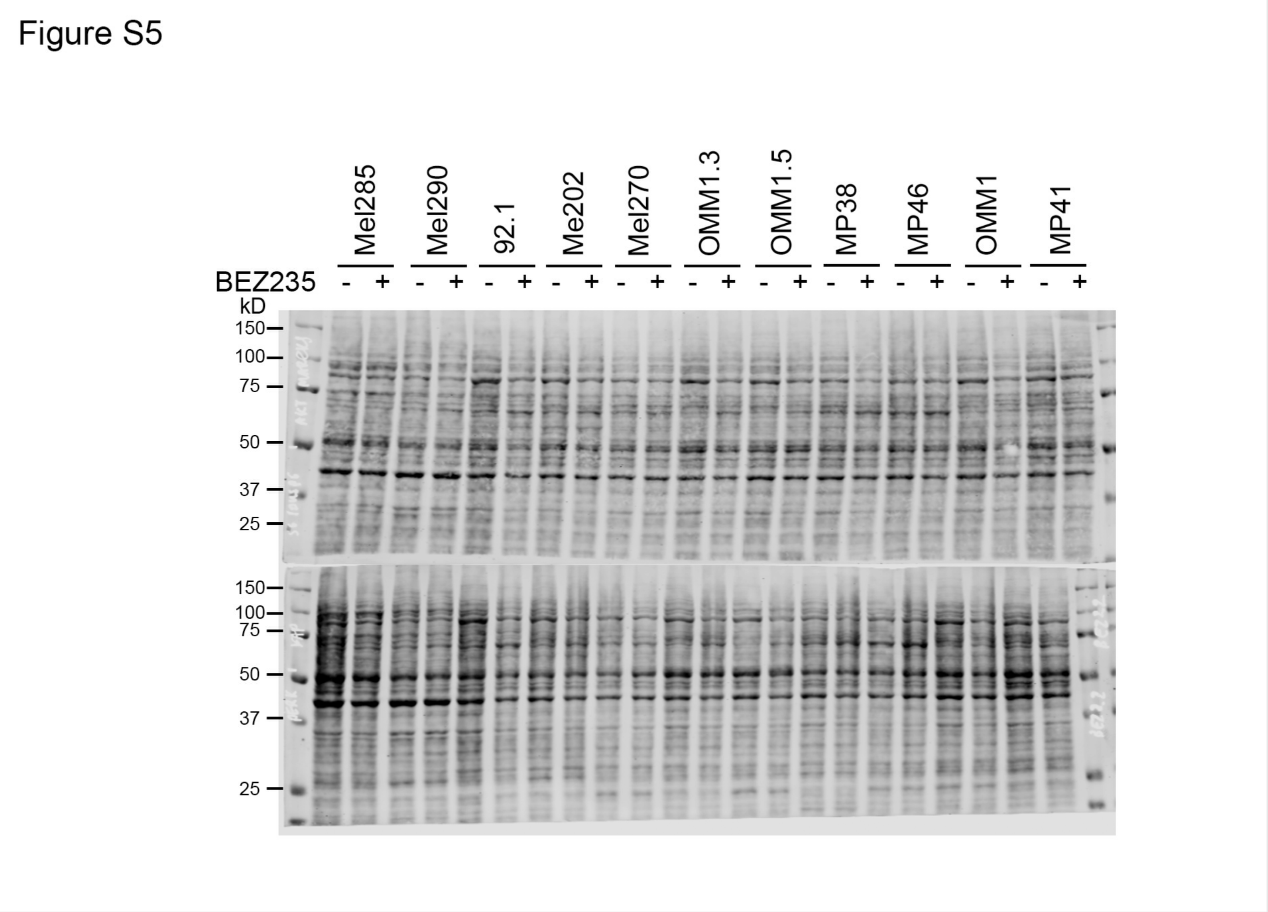

Figure S5: REVERT total protein stain

Blots showing REVERT total protein stain corresponding to Figure 3B. kD, kilodalton.

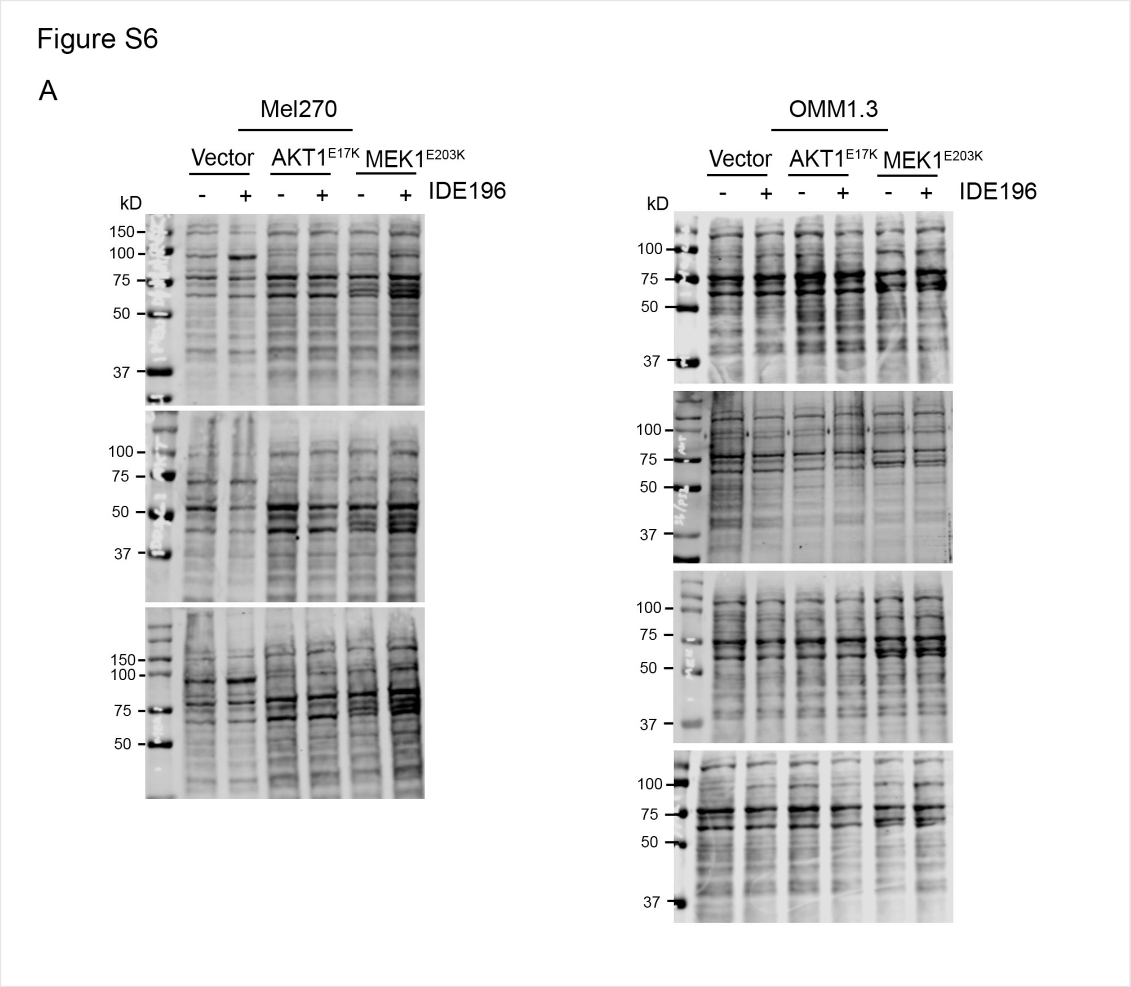

Figure S6: REVERT total protein stain

Blots showing REVERT total protein stain corresponding to Figure 4A. kD, kilodalton.

Figure S7

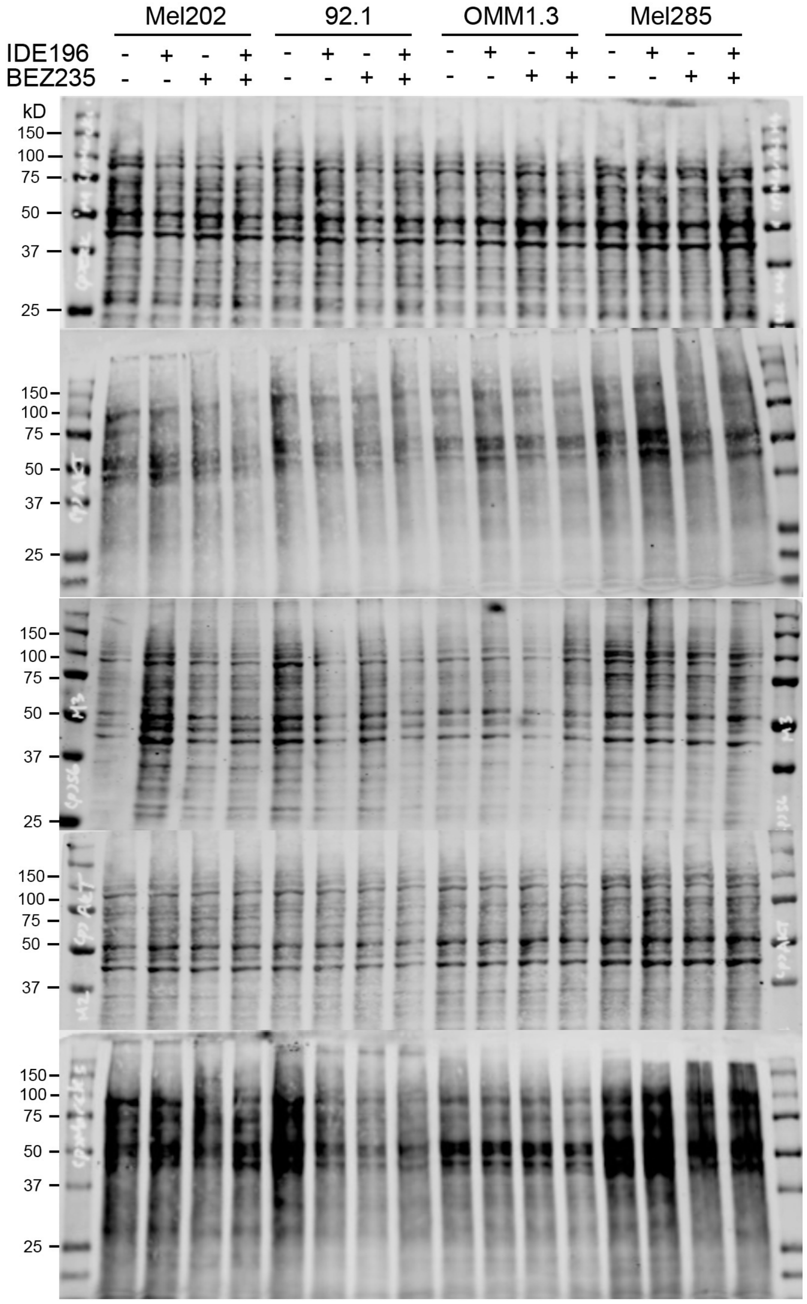

Figure S7: REVERT total protein stain

Blots showing REVERT protein stain corresponding to Figure 5A. kD, kilodalton.
