# Supplementary material for: PKC-independent PI3K signalling diminishes PKC inhibitor sensitivity in uveal melanoma
Source: Oncogenesis. 2024 Feb 28;13(1):9. doi: 10.1038/s41389-024-00511-8 (PMC10902289; doi:10.1038/s41389-024-00511-8)
Supplement: Supplementary file 2 — Supplementary Tables [file 41389_2024_511_MOESM2_ESM.pdf]

Table S1: Detailed characteristics of patients and tumour samples used in this study (subset of data derived from Park et al. Cancers 2021, 13:1740)

Pt ID	Age	Sex	ECOG PS	Mutation	Baseline LDH (U/L)	Site of disease	Primary tumour type	PRE sample site	Treatment	EDT sample site	Days from Treatment to EDT tissue sample	Best Response	PFS (mths)
4	69	F	0	$GNAQ^{R183Q}$	411	Liver, lung, LN	Iris	Liver	IDE196	Liver	15	SD ≥ 6 months	9.3
9	57	F	0	$GNA11^{Q209L}$	186	Liver	Unknown	Liver	IDE196	Liver	12	SD < 6 months	3.7
10	67	F	0	$CYSLTR2^{L129Q}$	233	Liver, bone, spleen	Choroidal	Liver	IDE196	Liver	15	PD	1.9
20	71	M	0	$GNAQ^{Q209P}$	192	Liver	Choroidal	Liver	IDE196	Liver	15	SD ≥ 6 months	6.1
21	62	M	0	$GNAQ^{Q209L}$	318	Liver, bone	Choroidal	Liver	IDE196	Liver	14	PD	3.5
12	67	F	1	$GNA11^{Q209L}$	389	Liver, lung, bone, LN	Choroidal	Liver	IDE196 + HDM201	Liver	26	PD	1.7
13	67	F	0	$GNAQ^{Q209P}$	225	Liver	Choroidal	Liver	IDE196 + HDM201	Liver	29	SD < 6 months	5.4
14	55	M	1	$GNAQ^{Q209P}$	454	Liver, lung, LN	Choroidal	Liver	IDE196 + HDM201	Liver	29	SD < 6 months	3.8
15	73	M	1	$GNAQ^{Q209P}$	835	Liver, lung, LN, spleen	Choroidal	Liver	IDE196 + HDM201	Liver	28	SD < 6 months	3.8
17	67	M	0	$GNAQ^{R183Q}$	869	Liver, spleen, bone	Choroidal	Liver	IDE196 + HDM201	Liver	27	SD < 6 months	3.9
24	65	F	0	$GNAQ^{Q209P}$	226	Liver	Choroidal	Liver	IDE196 + HDM201	Liver	28	SD < 6 months	4.2

Age at start of treatment shown
SD, stable disease; PD, progressive disease
Sex: M, male; F, female
Site of disease: LN, lymph node
PRE, baseline sample; EDT, early during treatment sample
LDH, lactate dehydrogenase
ECOG PS, Eastern Cooperative Oncology Group Performance Status
Patient #17 had a 3 day dose interruption during the PRE-EDT sample period and was on treatment at the time of EDT sample collection.
PFS, progression free survival

Table S2 Single sample geneset enrichment (ssGSEA) scores derived from the transcriptome data of patient matched PRE and EDT uveal melanoma biopsies

Patient ID	4	4	9	9	10	10	20	20	21	21	12	12	13	13	14	14	15	15
Biopsy timing	PRE	EDT	PRE	EDT	PRE	EDT	PRE	EDT	PRE	EDT	PRE	EDT	PRE	EDT	PRE	EDT	PRE	EDT
HALLMARK_TNFA_SIGNALING_VIA_NFKB	-326.97	-1214.04	-259.04	-328.26	-560.44	-698.45	-104.49	-399.84	392.16	73.04	1026.15	99.46	535.53	-206.43	-783.37	-961.66	1111.08	775.60
HALLMARK_HYPOXIA	2091.52	1555.28	1010.35	1595.82	822.98	1311.01	1907.34	1459.01	2172.38	1216.35	1938.10	1717.84	2034.32	2205.90	858.64	157.60	2440.65	2026.58
HALLMARK_CHOLESTEROL_HOMEOSTASIS	2376.13	4218.12	1868.82	3399.24	1227.57	3445.29	2666.66	3276.35	2813.86	1574.95	3299.86	2789.96	2944.38	3789.19	1553.67	938.07	2917.75	2473.76
HALLMARK_MITOTIC_SPINDLE	292.73	-762.72	-1020.52	-941.17	172.24	-1013.64	-818.54	-941.17	118.89	-216.87	87.84	-229.06	-391.40	-1050.06	-222.38	-121.82	90.60	-279.64
HALLMARK_WNT_BETA_CATENIN_SIGNALING	-283.76	-888.20	-466.86	-477.45	346.54	-643.57	-808.02	-568.21	-131.30	-138.47	-727.89	-992.91	-799.72	-1609.01	-137.60	26.97	-61.80	-347.25
HALLMARK_TGF_BETA_SIGNALING	-367.74	-697.79	-579.08	-373.66	-1077.12	-848.72	-106.74	70.51	604.43	772.37	541.40	-125.21	555.76	218.10	-549.88	-437.70	1404.71	628.60
HALLMARK_IL6_JAK_STAT3_SIGNALING	74.30	-333.63	-261.05	-611.47	-894.23	-359.52	601.39	682.21	-22.30	-80.03	2035.18	445.16	68.29	659.96	-1652.26	-1881.85	707.32	-298.24
HALLMARK_DNA_REPAIR	3328.29	1657.79	2218.61	1830.97	2189.47	1468.98	1704.04	1415.99	2874.60	2069.07	2176.65	2403.65	2902.24	1696.24	2406.71	2187.92	2304.96	2826.48
HALLMARK_G2M_CHECKPOINT	1493.56	-662.57	-1317.51	-1545.11	239.42	-1211.83	-318.50	-1375.40	580.26	-482.84	654.98	843.88	1080.37	-717.91	151.40	232.46	-528.95	-80.53
HALLMARK_APOPTOSIS	2526.52	1971.65	1719.73	2118.08	1616.70	2010.09	2060.11	2542.62	2693.51	1969.00	3274.98	2340.24	2490.79	2496.54	1274.60	747.30	3540.71	2682.45
HALLMARK_NOTCH_SIGNALING	456.21	-251.29	-89.07	-264.75	236.91	-330.68	-503.47	-426.48	1096.00	636.03	553.82	569.95	990.68	-56.07	-1196.85	-1201.86	885.06	455.37
HALLMARK_ADIPOGENESIS	3235.57	4820.45	3238.28	3928.41	2554.88	3908.47	4535.71	4525.50	3545.43	2701.60	4150.63	4350.47	4299.42	5016.18	2641.90	2325.25	3575.27	3875.12
HALLMARK_ESTROGEN_RESPONSE_EARLY	-1254.85	-453.27	-868.75	-2.53	-991.67	-362.38	-262.41	-418.66	-390.93	-700.90	-817.45	-824.95	-671.00	-221.81	-1075.76	-967.94	-423.66	-431.06
HALLMARK_ESTROGEN_RESPONSE_LATE	155.24	2633.94	-89.26	1830.40	-882.75	1782.83	2342.83	2549.55	205.63	-287.39	971.37	1226.68	1031.37	3019.27	-734.43	-718.63	197.20	778.53
HALLMARK_ANDROGEN_RESPONSE	2173.26	2821.02	2312.49	2682.82	1719.53	3369.70	3734.07	2333.70	2845.94	2367.71	4231.66	3587.07	2983.75	3767.40	623.16	509.35	4168.15	3173.67
HALLMARK_MYOGENESIS	1800.89	998.64	1150.64	1619.36	1118.80	1271.59	748.68	1381.57	2126.02	1803.49	1393.31	1416.23	1823.30	706.01	522.09	460.85	3089.88	2010.47
HALLMARK_PROTEIN_SECRETION	3833.33	1755.15	2919.48	2661.57	2575.42	2474.11	2898.17	1525.57	3986.75	3112.85	3814.31	3409.33	3625.68	2656.31	2478.45	2082.56	4074.76	3775.61
HALLMARK_INTERFERON_ALPHA_RESPONSE	4012.98	2167.05	3837.28	3225.90	3564.38	4268.66	3903.79	3129.72	4017.13	3587.30	5576.43	3574.61	3146.43	3165.98	504.26	63.96	5363.79	3451.79
HALLMARK_INTERFERON_GAMMA_RESPONSE	2884.55	1504.31	2816.86	2252.65	1959.80	3135.49	3135.84	2559.09	2734.70	2336.51	5061.78	2701.22	2013.97	2479.70	-631.78	-819.55	4448.10	2301.29
HALLMARK_APICAL_JUNCTION	1556.92	-61.07	563.14	578.86	833.58	344.48	12.31	866.72	1511.63	1127.28	1229.16	672.49	800.25	295.61	223.28	187.19	2029.00	908.44
HALLMARK_APICAL_SURFACE	1124.10	-1288.09	-444.72	-156.24	705.83	-495.34	-947.07	-555.56	730.45	351.04	1002.11	-381.89	-439.66	-1537.47	-800.82	-942.49	1430.09	-159.85
HALLMARK_HEDGEHOG_SIGNALING	-1345.52	841.89	-41.57	982.40	-1115.69	-364.72	221.36	665.25	-875.92	-893.14	-761.97	-941.82	-1347.68	-287.80	-930.30	-743.12	-1086.26	-938.78
HALLMARK_COMPLEMENT	944.30	4467.55	2205.29	3640.16	-130.91	3605.47	4259.93	4735.13	1636.80	920.43	4301.57	3345.35	2577.39	4869.44	329.10	6.70	2350.41	1650.05
HALLMARK_UNFOLDED_PROTEIN_RESPONSE	4300.57	2172.98	3602.09	2930.95	4023.27	3297.84	3433.48	2263.71	4295.15	3260.37	3980.12	4037.01	4275.79	3060.34	4137.80	3918.44	3915.78	4168.28
HALLMARK_PI3K_AKT_MTOR_SIGNALING	2899.95	1301.68	1683.33	1416.52	1708.15	1486.43	1566.99	1286.33	2829.67	1734.28	3219.61	1950.10	2344.16	1896.30	1557.27	1058.53	2829.30	1915.93
HALLMARK_MTORC1_SIGNALING	3962.24	2524.49	2636.93	2382.39	2359.98	2598.46	3171.98	1924.13	3965.90	2404.95	3927.62	3676.78	3993.89	2917.28	2587.60	1993.33	3455.21	3262.24
HALLMARK_E2F_TARGETS	2451.55	-650.06	-681.35	-1383.04	752.69	-1089.43	40.72	-1362.39	1108.68	-349.38	569.36	1566.34	1886.33	-1045.53	861.79	902.09	-466.90	641.03
HALLMARK_MYC_TARGETS_V1	6294.67	4391.81	5780.67	5077.47	6245.37	5588.65	5835.08	4274.78	6320.39	5620.39	6109.78	6301.29	6407.76	4944.72	6348.13	6230.25	5935.07	6224.63
HALLMARK_MYC_TARGETS_V2	3573.71	1086.70	2304.66	1468.84	3334.00	1780.80	2027.29	517.05	3298.28	1468.77	2296.73	2651.70	3204.79	1135.34	3227.09	3087.83	2105.57	2715.35
HALLMARK_EPITHELIAL_MESENCHYMAL_TRANSITION	1351.29	-361.15	933.94	1387.93	202.23	338.19	615.89	1410.64	2725.62	2233.89	2365.15	2154.17	2224.20	1378.57	-2.09	-191.48	3488.20	2165.36
HALLMARK_INFLAMMATORY_RESPONSE	-914.01	-1544.61	-930.18	-1308.93	-1683.46	-958.27	-779.76	-573.05	-897.45	-743.52	582.67	-910.47	-551.87	-342.72	-2424.71	-2458.34	311.27	-924.13
HALLMARK_XENOBIOTIC_METABOLISM	-741.00	5856.16	1359.71	4669.59	-1275.09	4614.61	5040.66	5593.77	-441.84	-997.10	3430.02	3352.57	1795.48	5802.36	-698.93	-900.72	-408.27	1013.73
HALLMARK_FATTY_ACID_METABOLISM	2077.87	4815.06	1983.18	3746.62	1162.78	3921.86	4100.71	4086.65	1999.67	1047.46	3388.60	3388.32	3132.99	4869.40	1068.63	898.92	2316.24	2495.76
HALLMARK_OXIDATIVE_PHOSPHORYLATION	5797.01	5470.25	5403.90	5315.45	5255.34	5617.79	5986.03	5062.44	5868.46	4912.57	5596.35	5991.37	6285.51	5732.66	5263.17	4869.98	5757.55	6107.05
HALLMARK_GLYCOLYSIS	2525.54	2346.58	1531.27	2083.32	1349.58	2136.47	2567.40	1811.07	2882.12	1603.28	2508.70	2660.85	2844.77	2524.84	1408.34	791.99	2362.83	2446.18
HALLMARK_REACTIVE_OXYGEN_SPECIES_PATHWAY	5132.59	5419.14	4527.40	5066.24	4298.05	5128.57	5217.44	5183.23	5190.89	4430.89	5794.28	5343.32	5265.09	5261.35	3696.70	2787.67	5229.36	4951.71
HALLMARK_P53_PATHWAY	2961.50	1927.48	2749.49	2513.27	2991.91	2583.22	2640.86	2178.00	3041.05	2308.78	2889.48	3143.93	3249.41	2819.93	2742.02	2434.66	3276.18	4206.55
HALLMARK_UV_RESPONSE_UP	2220.47	1496.29	1764.36	2245.18	1825.62	2313.43	2122.56	1836.56	2264.82	1471.54	2666.92	1994.02	2459.61	2067.94	1558.13	1245.92	2257.22	1994.29
HALLMARK_UV_RESPONSE_DN	-603.45	-793.81	-743.18	-372.47	-674.67	-501.38	-138.42	-376.52	128.73	147.24	-277.75	-699.64	-150.21	-118.74	-729.93	-508.77	758.69	110.29
HALLMARK_ANGIOGENESIS	1192.15	3577.33	1349.67	3670.30	-1264.31	2733.54	3087.86	3873.48	2806.57	2242.13	3345.54	3050.54	2772.01	4399.37	-1286.24	-1105.59	3825.04	2911.25
HALLMARK_HEME_METABOLISM	899.46	2790.37	1610.45	2263.96	845.25	1732.53	2402.03	2869.69	1138.22	1057.33	2190.18	1993.91	1718.54	2587.72	658.60	569.38	1446.85	1468.78
HALLMARK_COAGULATION	885.50	6216.31	3839.84	5830.09	-1114.17	5545.63	5921.89	6280.12	1618.08	792.00	5096.86	5133.70	3849.91	6354.35	678.57	128.09	2269.13	2928.54
HALLMARK_IL2_STAT5_SIGNALING	1535.76	219.26	725.87	880.07	486.01	812.03	1067.68	653.16	1570.32	852.50	1788.97	1077.08	1583.31	987.41	119.21	-310.72	2380.03	1414.76
HALLMARK_BILE_ACID_METABOLISM	-1354.39	5590.78	231.32	4052.49	-1607.73	4126.20	4548.77	4959.25	-1400.69	-1565.35	1752.94	1951.26	758.77	5496.65	-1506.27	-1608.87	-869.68	-17.79
HALLMARK_PEROXISOME	2437.05	6103.88	3211.06	5409.87	1362.31	5808.19	6131.86	5832.11	2269.72	1656.96	4429.75	4248.51	3741.60	6141.83	1293.91	1057.32	2212.64	2779.78
HALLMARK_ALLOGRAFT_REJECTION	3840.16	612.34	3711.11	2448.12	3608.19	3730.14	3982.67	2323.31	3736.98	3351.73	5379.83	4681.92	4619.91	2725.71	3060.30	3324.86	4851.11	4361.06
HALLMARK_SPERMATOGENESIS	-1053.89	-1791.23	-2730.27	-2386.89	-1377.26	-2229.01	-1803.72	-2034.87	-1425.04	-2045.54	-2081.62	-1467.28	-1173.11	-1823.50	-796.14	-1046.01	-1802.52	-1501.31
HALLMARK_KRAS_SIGNALING_UP	-1751.15	950.99	-875.70	204.98	-2068.89	118.04	913.68	1331.10	-1297.24	-1013.41	1077.06	619.39	-263.17	1961.70	-2290.00	-2226.27	-375.55	-153.75
HALLMARK_KRAS_SIGNALING_DN	-2302.13	-16.15	-1868.83	-437.90	-2090.50	-347.78	-626.01	327.75	-2179.27	-2398.16	-1466.72	-1931.76	-2656.20	491.42	-2271.67	-2072.97	-2438.84	-2511.76
HALLMARK_PANCREAS_BETA_CELLS	-595.97	-224.72	-1602.25	-1021.26	-2249.77	-749.55	484.25	-927.84	-248.84	-1335.83	-268.75	141.64	1120.92	1010.55	-1031.10	-1933.91	312.30	401.98
MAPK_UP#2	-1396.16	-1577.17	-1173.93	-1067.57	-1530.24	-1499.10	-911.82	-1016.02	-523.05	-1520.00	-833.22	-1066.51	-273.23	-1315.36	-1307.01	-1552.19	220.29	358.93
MAPK_UP#1	-668.96	-2185.80	-6.47	-756.00	-1049.15	-1303.67	-356.46	-1725.44	644.46	-1261.39	-764.02	-362.93	698.50	-1682.50	-58.25	-221.16	281.82	979.99
YAP1_UP	2504.13	392.31	1195.97	831.33	1448.19	542.56	788.76	167.30	2422.62	1672.60	1571.38	1250.95	2206.37	629.65	1673.35	1151.67	2914.89	1849.48

PRE, pre-treatment; EDT, early during treatment

MAPK_UP#1 signature derived from Pratilas et al. 2009. Cancer Res 68:9375

MAPK_UP#2 signature derived from Nazarian et al. 2010. Nature 468:973

YAP1_UP signature derived from Molecular Signatures Databse c6 v2023.1

17	17	24	24			
PRE	EDT	PRE	EDT		Median PRE	Median EDT
130.51	-176.30	227.19	-408.57		130.51	-328.26
1500.01	1248.62	1295.57	1886.17		1907.34	1555.28
2283.70	2166.76	2285.02	4075.04		2376.13	3276.35
-537.73	82.13	-1011.79	-1027.77		-222.38	-762.72
-831.59	-190.64	-514.21	-787.93		-466.86	-568.21
-534.21	152.23	-361.31	-434.01		-361.31	-125.21
-76.45	-237.94	417.29	614.84		68.29	-237.94
2402.91	2160.87	2029.51	1677.06		2304.96	1830.97
556.43	-205.17	-411.89	-411.00		239.42	-482.84
2064.87	2221.95	2233.98	2595.67		2233.98	2221.95
38.68	193.73	-898.33	-659.46		236.91	-251.29
3641.33	2870.65	3541.47	4760.49		3545.43	3928.41
-545.46	-336.25	-783.67	45.21		-783.67	-418.66
492.67	-227.41	-38.72	2679.55		197.20	1782.83
1989.69	1873.01	2675.63	3601.30		2675.63	2821.02
1602.39	2110.28	778.40	1518.13		1393.31	1416.23
3121.36	3200.26	2748.98	2758.27		3121.36	2661.57
920.61	1195.62	3753.52	4108.51		3837.28	3225.90
88.42	38.24	2795.30	3107.93		2795.30	2336.51
495.47	684.16	290.42	65.80		800.25	578.86
-168.25	833.59	-121.97	-343.53		-121.97	-381.89
-1626.30	-967.87	-760.76	330.34		-930.30	-364.72
1354.17	-100.61	2592.10	4448.43		2205.29	3605.47
4339.15	4278.46	4519.60	3640.83		4137.80	3297.84
1114.64	1323.78	1883.23	1854.99		1883.23	1486.43
3182.11	2232.68	3076.62	3256.93		3182.11	2524.49
1705.04	352.42	79.93	-497.21		752.69	-497.21
6380.90	6219.19	6376.42	5865.04		6294.67	5620.39
3168.41	2459.21	3287.53	2587.89		3204.79	1780.80
1754.52	1468.22	1277.88	745.24		1351.29	1387.93
-1732.20	-2323.66	-14.74	-707.56		-897.45	-924.13
1443.67	-1083.67	2112.98	5565.16		1359.71	4614.61
2449.93	1364.67	2439.40	4557.09		2316.24	3746.62
5936.52	5297.85	5553.50	5703.47		5757.55	5470.25
2391.67	1727.84	1704.75	2776.17		2391.67	2136.47
4324.07	4232.79	4799.87	5353.67		5132.59	5128.57
3092.01	2986.59	3352.22	2601.20		2991.91	2583.22
2084.67	1640.38	2027.69	2476.17		2122.56	1994.02
44.24	435.42	-553.04	-157.69		-277.75	-372.47
824.65	-766.47	1387.29	3757.05		1387.29	3050.54
1339.74	1157.69	1884.42	2465.31		1446.85	1993.91
3479.89	-45.40	4041.45	6135.95		3479.89	5545.63
728.06	477.43	1258.42	1182.87		1258.42	852.50
548.17	-1389.16	785.77	5183.16		231.32	4052.49
3406.96	1609.66	3632.87	6043.45		3211.06	5409.87
3827.14	3535.22	4973.95	3132.27		3840.16	3324.86
-792.75	-1250.13	-1184.22	-1352.17		-1377.26	-1791.23
-1045.42	-1782.00	-15.37	1279.34		-875.70	204.98
-2583.52	-2336.99	-2021.69	24.04		-2179.27	-437.90
-190.02	-1360.16	-618.13	366.36		-268.75	-749.55
-555.82	-1759.29	-814.94	-1167.89		-833.22	-1315.36
329.25	-1307.88	-234.62	-711.57		-58.25	-1261.39
1823.50	2163.75	1422.19	814.31		1673.35	831.33

Table S3 Pearson correlation values between the Hallmark_MYC_TARGETS_V2 with proliferation signature and other genesets

Geneset	Pearson correlation	P-value	FDR(BH)
HALLMARK_MYC_TARGETS_V2	1	0	0.01
HALLMARK_MYC_TARGETS_V1	0.92	0	0.01
HALLMARK_UNFOLDED_PROTEIN_RESPONSE	0.91	0	0.01
HALLMARK_E2F_TARGETS	0.83	0	0.01
HALLMARK_G2M_CHECKPOINT	0.75	0	0.01
HALLMARK_DNA_REPAIR	0.75	0	0.01
HALLMARK_SPERMATOGENESIS	0.71	0	0.01
MAPK_UP#1	0.67	0	0.01
YAP1_UP	0.64	0	0.01
HALLMARK_P53_PATHWAY	0.57	0	0.01
HALLMARK_ALLOGRAFT_REJECTION	0.55	0.01	0.02
HALLMARK_MITOTIC_SPINDLE	0.52	0.01	0.02
HALLMARK_PROTEIN_SECRETION	0.48	0.03	0.06
HALLMARK_MTORC1_SIGNALING	0.46	0.03	0.06
HALLMARK_WNT_BETA_CATENIN_SIGNALING	0.41	0.07	0.12
HALLMARK_APICAL_SURFACE	0.36	0.11	0.18
HALLMARK_OXIDATIVE_PHOSPHORYLATION	0.31	0.2	0.31
HALLMARK_PI3K_AKT_MTOR_SIGNALING	0.29	0.18	0.28
MAPK_UP#2	0.19	0.44	0.59
HALLMARK_TNFA_SIGNALING_VIA_NFKB	0.18	0.43	0.59
HALLMARK_APICAL_JUNCTION	0.16	0.47	0.61
HALLMARK_UV_RESPONSE_UP	0.11	0.59	0.68
HALLMARK_IL2_STAT5_SIGNALING	0.11	0.58	0.68
HALLMARK_NOTCH_SIGNALING	0.09	0.71	0.77
HALLMARK_EPITHELIAL_MESENCHYMAL_TRANSITION	0.04	0.84	0.86
HALLMARK_MYOGENESIS	0.04	0.82	0.85
HALLMARK_GLYCOLYSIS	-0.02	0.96	0.96
HALLMARK_UV_RESPONSE_DN	-0.07	0.74	0.79
HALLMARK_PANCREAS_BETA_CELLS	-0.09	0.68	0.75
HALLMARK_HYPOXIA	-0.13	0.65	0.73
HALLMARK_TGF_BETA_SIGNALING	-0.14	0.57	0.68
HALLMARK_INTERFERON_ALPHA_RESPONSE	-0.16	0.5	0.63
HALLMARK_APOPTOSIS	-0.16	0.53	0.65
HALLMARK_INFLAMMATORY_RESPONSE	-0.24	0.32	0.44
HALLMARK_INTERFERON_GAMMA_RESPONSE	-0.25	0.28	0.4
HALLMARK_IL6_JAK_STAT3_SIGNALING	-0.29	0.21	0.31
HALLMARK_ANDROGEN_RESPONSE	-0.35	0.12	0.2
HALLMARK_REACTIVE_OXYGEN_SPECIES_PATHWAY	-0.35	0.1	0.17
HALLMARK_ADIPOGENESIS	-0.51	0.01	0.03
HALLMARK_CHOLESTEROL_HOMEOSTASIS	-0.54	0.01	0.02
HALLMARK_ESTROGEN_RESPONSE_EARLY	-0.54	0	0.01
HALLMARK_FATTY_ACID_METABOLISM	-0.59	0	0.01
HALLMARK_COAGULATION	-0.6	0	0.01
HALLMARK_ANGIOGENESIS	-0.61	0	0.01
HALLMARK_PEROXISOME	-0.62	0	0.01
HALLMARK_XENOBIOTIC_METABOLISM	-0.65	0	0.01
HALLMARK_COMPLEMENT	-0.65	0	0.01
HALLMARK_KRAS_SIGNALING_UP	-0.66	0	0.01
HALLMARK_ESTROGEN_RESPONSE_LATE	-0.67	0	0.01
HALLMARK_BILE_ACID_METABOLISM	-0.69	0	0.01
HALLMARK_HEME_METABOLISM	-0.7	0	0.01
HALLMARK_HEDGEHOG_SIGNALING	-0.73	0	0.01
HALLMARK_KRAS_SIGNALING_DN	-0.74	0	0.01

FDR(BH), P-value adjusted for false discovery rate (Benjamini Hochberg method)
MAPK_UP#1 signature derived from Pratilas et al. 2009. Cancer Res 68:9375
MAPK_UP#2 signature derived from Nazarian et al. 2010. Nature 468:973
YAP1_UP signature derived from Molecular Signatures Databse c6 v2023.1

Table S4 Characteristics and treatment response data for uveal melanoma cells

		1µM IDE196[a]		5µM IDE196[a]		5nM Trametinib		10nM Trametinib		500nM BEZ235		2000nM BEZ235	
Cell Lines	Genotype	Change in % sub-G1	% S-phase inhibition	Change in % sub-G1	% S-phase inhibition	Change in % sub-G1	% S-phase inhibition	% cell death	% S-phase inhibition	Change in % sub-G1	% S-phase inhibition	Change in % sub-G1	% S-phase inhibition
Mel285	-	0.0	2.1	-0.2	8.7	-0.5	28.7	-0.1	25.2	0.0	9.0	0.1	27.2
Mel290	-	0.0	13.4	0.1	16.9	0.0	34.6	0.0	44.1	0.0	89.3	0.0	90.3
92.1	GNAQ Q209L	3.2	87.0	14.8	95.9	0.1	29.5	1.0	42.8	1.1	52.5	5.1	86.9
Mel202	GNAQ Q209L & R210K	1.3	61.5	3.4	64.4	0.0	42.1	0.3	53.9	0.8	69.0	2.3	82.4
Mel270	GNAQ Q209P	65.0	78.2	60.5	72.2	18.4	64.2	33.6	63.5	13.5	74.8	19.6	71.0
OMM1.3	GNAQ Q209P	8.4	83.9	3.6	91.4	0.2	36.7	0.2	45.9	0.5	76.3	0.8	77.9
OMM1.5	GNAQ Q209P	2.6	85.0	2.6	88.0	0.0	30.8	0.0	41.6	0.1	19.9	0.4	32.7
MP38	GNAQ Q209L	0.4	52.3	0.9	71.2	0.3	59.4	0.6	74.6	0.6	63.5	0.9	52.4
MP46	GNAQ Q209L	1.9	50.1	1.7	60.1	0.3	5.0	0.7	15.1	0.8	19.1	0.9	13.0
OMM1	GNA11 Q209L	36.6	22.4	50.2	46.5	3.7	14.5	12.7	21.6	59.9	6.5	59.6	17.4
MP41	GNA11 Q209L	1.5	82.3	2.2	87.4	0.0	7.4	-0.1	18.4	0.2	-2.8	0.4	2.8

[a]Data derived from Park et al. 2020 Cancer Gene Ther 29:1384-1393

Data are the average of at least three biological replicates

% S-phase inhibition = (percentage of S-phase in the control-treated cells – percentage of S-phase in drug-treated cells) / (percentage of S-phase in the control-treated cells) x100

Change in % sub-G1 = % sub G1 in drug-treated cells - % sub-G1 in control-treated cells

- no detectable mutation

Table S5 Synergy parameters and CI values for UM cell lines treated with a combination of IDE196 and BEZ235

	IDE196 + BEZ235 (4:1)					
	Parameters			CI values at		
	D_m	m	r	ED_{50}	ED_{75}	ED_{90}
**MEL202**	3.31644	1.50540	0.97955	**0.07990**	**0.04366**	**0.02699**
**92.1**	2.96453	1.2727	0.99679	**0.26926**	**0.26142**	**0.2549**
**OMM1**	0.13446	0.47612	0.84951	**0.06236**	**0.01187**	**0.01286**
**MP41**	10.0056	0.88891	0.99485	**0.24898**	**0.34308**	**0.47343**

Data shown were derived from average sub-G1 drug-treated – average sub G1 vehicle-treated cells; 3-4 independent experiments per cell line

Dm, median effective dose; **m**, slope; **r**, linear regression coefficient; **CI**, combination index

ED_{50}, dose effective in 50% of the population; **ED_{75}**, dose effective in 75% of the population;

ED_{90}, dose effective in 90% of the population

Median effect curves for OMM13, Mel290, Mel285 had negative slopes and could not be analysed for synergy
